# Anti-Inflammatory Ergosteroid Derivatives from the Coral-Associated Fungi *Penicillium oxalicum* HL-44

**DOI:** 10.3390/molecules28237784

**Published:** 2023-11-26

**Authors:** Cheng Pang, Yu-Hong Chen, Hui-Hui Bian, Jie-Ping Zhang, Li Su, Hua Han, Wen Zhang

**Affiliations:** 1 School of Pharmaceutical Sciences, Zhejiang Chinese Medical University, Gao-Ke Rd., Hangzhou 311402, China; 2 School of Medicine, Tongji University, 1238 Gonghexin Rd., Shanghai 200070, China; 3 Institute of Translational Medicine, Shanghai University, 99 Shangda Rd., Shanghai 200444, China

**Keywords:** *Penicillium oxalicum*, coral-associated fungi, ergosteroid derivatives, anti-inflammatory activity

## Abstract

To obtain the optimal fermentation condition for more abundant secondary metabolites, Potato Dextrose Agar (PDA) medium was chosen for the scale-up fermentation of the fungus *Penicillium oxalicum* HL-44 associated with the soft coral *Sinularia gaweli*. The EtOAc extract of the fungi HL-44 was subjected to repeated column chromatography (CC) on silica gel and Sephadex LH-20 and semipreparative RP-HPLC to afford a new ergostane-type sterol ester (**1**) together with fifteen derivatives (**2**–**16**). Their structures were determined with spectroscopic analyses and comparisons with reported data. The anti-inflammatory activity of the tested isolates was assessed by evaluating the expression of pro-inflammatory factors *Tnfα* and *Ifnb1* in Raw264.7 cells stimulated with LPS or DMXAA. Compounds **2**, **9**, and **14** exhibited significant inhibition of *Ifnb1* expression, while compounds **2**, **4**, and **5** showed strong inhibition of *Tnfα* expression in LPS-stimulated cells. In DMXAA-stimulated cells, compounds **1**, **5**, and **7** effectively suppressed *Ifnb1* expression, whereas compounds **7**, **8**, and **11** demonstrated the most potent inhibition of *Tnfα* expression. These findings suggest that the tested compounds may exert their anti-inflammatory effects by modulating the cGAS-STING pathway. This study provides valuable insight into the chemical diversity of ergosteroid derivatives and their potential as anti-inflammatory agents.

## 1. Introduction

Marine organisms are a rich source of steroids with potent anti-inflammatory activity. They perform functions by attenuating the activity of the immune system and suppressing inflammation [1]. The ergosteroids are the vast majority of these steroids and construct the main steroid of fungi [2].

*Penicillium oxalicum* is a frequently isolated fungus exhibiting a wide spectrum of physiological activities that are of relevance in agriculture, biotechnology, food quality assessments, and medicine [3]. Previous chemical investigations of *P. oxalicum* led to the isolation of alkaloids [4,5], polyketides [6,7], meroterpenoids [8,9], and steroids [10,11], exhibiting bioactivities of brine shrimp lethality and anti-*Rhizoctonia Solani*, anti-neuroinflammatory, antipancreatic tumor, anti-HAB (harmful algal bloom), antiviral, and antibacterial properties. *P. oxalicum* is known for its ability in biotransformation. The fungi were found to be able to act as hyperproducers of chitin deacetylase for converting chitin to chitosan, transforming protopanaxadiol-type saponins to ginsenoside compound K, and promoting the biotransformation of ethinylestradiol 1 [12,13].

Research conducted in our group rests on the chemical and pharmacological investigation of secondary metabolites from marine invertebrates and associated fungi. Recently, we focused on the molecules having immunomodulatory and neuronal modulatory activities. A drimane meroterpenoid characterized by a thioglycerate moiety [14] and a drimane meroterpenoid with a unique borate ring system [15] were obtained from the fungi *Alternaria* sp. ZH-15 associated with the soft coral *Lobophytum crassum* collected from the Dongsha Atoll in the South China Sea. These compounds displayed potential as novel anti-epileptic agents due to their significant inhibitory activities of spontaneous synchronous Ca^2+^ oscillations (SCO) and 4-aminopyridine-induced epileptic discharges in the low micromolar concentration range. Sixteen 9,10-secosteroids were isolated from the gorgonian *Verrucella umbraculum* collected from the Xisha islands in the South China Sea. These compounds exhibited significant suppressive effects on CD4^+^ T lymphocyte cell differentiation in an in vitro bioassay, representing the first report of 9,10-secosteroids to exhibit immunomodulation activity [16]. As part of ongoing screening for bioactive metabolites from China marine sources, a fungus strain of *P*. *oxalicum* HL-44 was isolated from the soft coral *Sinularia gaweli* collected from the Xisha area of the South China Sea. Chemical investigation of this fungi led to the isolation of a new ergostane-type sterol ester (**1**) and fifteen known derivatives (**2**–**16**). Their structures were determined with extensive spectroscopic analyses and comparisons with reported data. We investigated the anti-inflammatory activities of these isolates in vitro by examining their effects on the expression of proinflammatory cytokines *Tnfα* and *Ifnb1* in Raw264.7 cells stimulated with LPS or DMXAA. In the present study, we describe the isolation, structure elucidation, and anti-inflammatory activities of these compounds.

## 2. Results and Discussion

To obtain the optimal fermentation condition, UPLC-MS was employed for the chemical analysis of fungal metabolites produced with strain HL-44 in five candidates of media, including Czapek Dox Agar (CZA) medium, Glucose Peptone Yeast (GPY) medium, PDA medium, Rose Bengal Medium (RBM), and Rice medium. The Total Ion Chromatography (TIC) of the PDA medium showed more abundant metabolites with ion peaks (*m*/*z*) in a mass spectrum ranging from 408 to 688. In particular, the ion peak at *m*/*z* 663.534 was fairly interesting. Thus, the PDA medium was chosen for the scale-up fermentation of the fungi HL-44.

**Figure 1:**
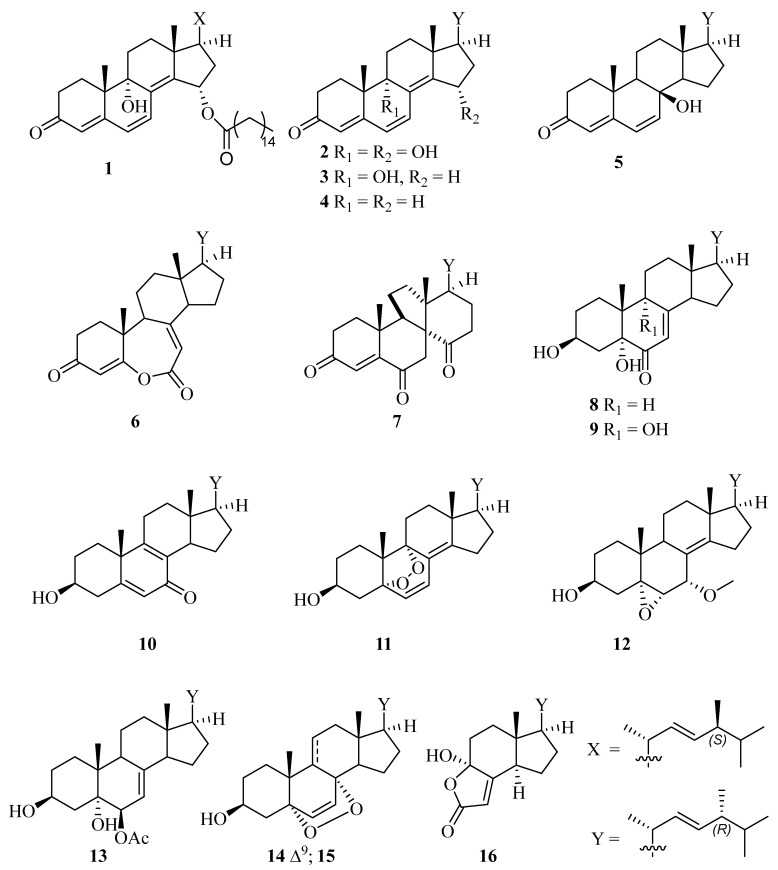
Structures of compounds **1**–**16** isolated from the fungi *P. oxalicum* HL-44**,** having a corresponding *β*-methyl in X or *α*-methyl in Y.

The fungal strain was then cultivated on the PDA medium for scale fermentation at 28 °C for 28 days and was then extracted ultrasonically with EtOAc to afford a residue after removal of the solvent under reduced pressure [14,15]. The crude extract was subjected to column chromatography (CC) silica gel, Sephadex LH-20, and reversed phase HPLC to afford compounds **1**–**16** (Figure 1). On the basis of spectroscopic techniques (^1^H-NMR, ^13^C-NMR) and comparison with data recorded in the references, compounds **2**–**16** were determined as (22*E*,24*R*)-9*α*,15*α*-dihydroxyergosta-4,6,8(14),22-tetraen-3-one (**2**) [17], ganodermaside D (**3**) [18], (22*E*,24*R*)-ergosta-4,6,8(14),22-tetraen-3-one (**4**) [19], isocyathisterol (**5**) [20], herbarulide (**6**) [21], dankasterones A (**7**) [22], (22*E*,24*R*)-ergosta-7,22-dien-3*β*,5*α*-diol-6-one (**8**) [23], (22*E*,24*R*)-ergosta-7,22-dien-3*β*,5*α*,9*α*-trihydroxy-6-one (**9**) [24], (22*E*,24*R*)-3*β*-hydroxyergosta-5,8,22-trien-7-one (**10**) [25], (22*E*,24*R*)-5*α*,9*α*-epidioxyergosta-6,8(14),22-triene-3*β*-ol (**11**) [26], (22*E*, 24*R*)-7*α*-methoxy-5*α*,6*α*-epoxyergosta-8(14),22-dien-3*β*-ol (**12**) [27], (22*E*,24*R*)-6-acetoxy-ergosta-7,22-dien-3*β*,5*α*,6*β*-triol (**13**) [28], (22*E*,24*R*)-5*α*,8*α*-epidioxyergosta-6,9(11),22-trien-3*β*-ol (**14**) [29], (22*E*,24*R*)-5*α*,8*α*-epidioxyergosta-6,22-dien-3*β*-ol (**15**) [30], and demethylincisterol A3 (**16**) [31].

**Figure 2:**
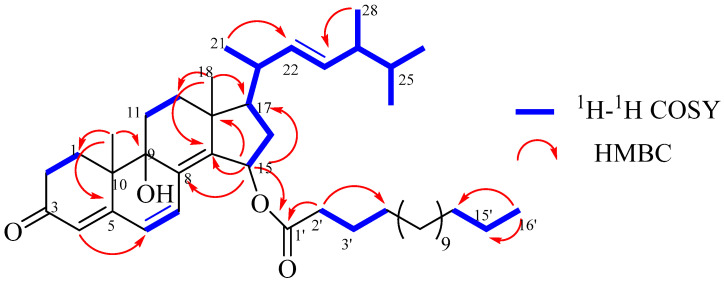
Key ^1^H-^1^H COSY and HMBC correlations of compound **1**.

**Figure 3:**
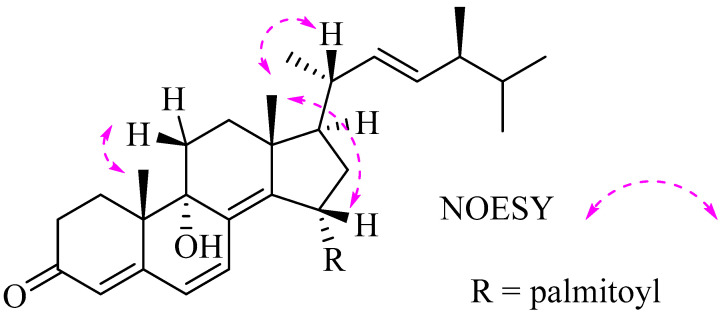
Key NOESY correlations of compound **1**.

Compound **1** was obtained as an optically yellowish oil. The HRESIMS gave a molecular formula as C_44_H_70_O_4_ on the basis of its pseudomolecular ion peak at *m*/*z* 663.53448 ([M + H]^+^), indicating 10 degrees of unsaturation. The IR spectrum revealed the presence of a hydroxy group (3344 cm^−1^) and carbonyl (1733 cm^−1^) functionalities. The characteristic IR absorptions at 1664 and 1595 cm^−1^ and the strong UV absorptions at 265 and 330 nm indicated the presence of a large conjugated carbonyl system in this molecule. These observations were in agreement with the NMR data for an oxygenated methine (*δ*_H_ 5.81; *δ*_C_ 71.8, CH), a tertiary oxygenated carbon atom (*δ*_C_ 72.8, C), four pairs of double bonds, one ester carbonyl atom (*δ*_C_ 173.6), and one ketone carbonyl atom (*δ*_C_ 199.3), taking into account six degrees of unsaturation. The remaining four degrees of unsaturation were due to the ring system in the molecule. Compound **1** resembled **2** in the NMR data (Table 1) except for signals for a palmitoyl moiety, which was supported by the 2D NMR analyses, as shown in Figure 2. The palmitoyl moiety was assigned at C-15 by the distinct HMBC effects of H-15 with C-8, C-13, C-17, and the ester carbonyl atom (C-1’). Compound **1** displayed the same relative configuration as that of **2** in the core structure, as shown in Figure 3. The *β*-configuration of H-15 was indicated by its NOE correlation with H_3_-18. The configuration at C-9 was suggested by comparing its shift value (*δ*_C_ 72.8, C) to those reported in the literature (*δ*_C_ 72.7/72.8, C) [17,32], indicating that the hydroxy group at C-9 was in the *α*-configuration. The absolute configuration of C-24 was assigned as *S* in **1** vs *R* in **2** based on the ^13^C NMR shift values of C-28. The NMR data for C-28 are reported at *δ* 17.6 ± 0.1 ppm for 24*R*-isomers and *δ* 18.0 ± 0.1 ppm for 24*S*-isomers [16,33]. The presence of a palmitic acid residue was proven with an MS analysis and comparison of the spectroscopic data with those reported data [34]. The existence of the fatty acyl moiety was indicated by the characteristic ^13^C signals for the ester carbonyl carbon (*δ*_C_ 173.6) and ^1^H signals for triplet methylene at *δ*_H_ 2.30, multiplet methylenes at *δ*_H_ 1.23–1.30, and a terminal methyl group at *δ*_H_ 0.88. The structure of **1** was thus determined as (22*E*,24*S*)-9*α*,15*α*-dihydroxyergosta-4,6,8(14),22-tetraen-3-one 15-palmitate, showing a 24*S* configuration vs. 24*R* in **2**. The compound is also characterized by a 15-palmitoyl moiety with respect to a hydroxyl group in **2**.

Derivatives of 15*α*-hydroxy steroids serve as key intermediates in the production of contraceptives [35,36]. A P450 enzyme, which is composed of the cytochrome P450 hydroxylase and the NADPH-cytochrome P450 reductase (CPR), has been reported to be associated with the 15*α*-hydroxylation reaction in *P. raistrickii* [37]. *P. raistrickii*-mediated 15*α*-hydroxylation of D-ethylgonendione is a key step for the production of gestodene [38]. The 9,15-hydroxylated ergosteroid **2** was only reported in *Omphalia lapidescens* [32] and *Ganoderma resinaceum* [17]. The isolation of **1** and **2** suggests that *P. oxalicum* strains have a potential in fungal transformation to afford 15*α*-hydroxyergosteroid. Compounds **3** and **4** have a chemical feature of conjugated 4,6,8-trien-3-one and have never been reported in *P. oxalicum*. Compound **6** is a ketodivinyllactone steroid with an unprecedented homo-6-oxaergostane skeleton isolated from the endophytic fungus *Pleospora herbarum* [39] with a structure revision at C24 with a chemical synthesis [21]. Compound **7** was an unprecedented steroid possessing a 13(14→8)*abeo*-8-ergostane skeleton first found in the Halichondria sponge-derived fungus *Gymnacella dankaliensis* [22]. Compound **11**, which features an unusual 1,2-dioxolane moiety, was only reported in *G. capense* [26] and *G. lingzhi* [40]. Although 5*α*,8*α*-epidioxysterols with variations in the side chains were most commonly reported from a number of sources, rare 5*α*,9*α*-epidioxy steroids were mainly isolated from different edible mushrooms [41,42]. The compound showed weak anti-HIV activity and remarkable cytotoxicity against A549 and MCF-7 tumor cell lines. Compound **16** is a highly degraded sterol, and its basic skeleton derives from a dramatic oxidative degradation of the sterol nucleus with the loss of all six carbon atoms of the A ring and the 19-methyl group [43].

**Figure 4:**
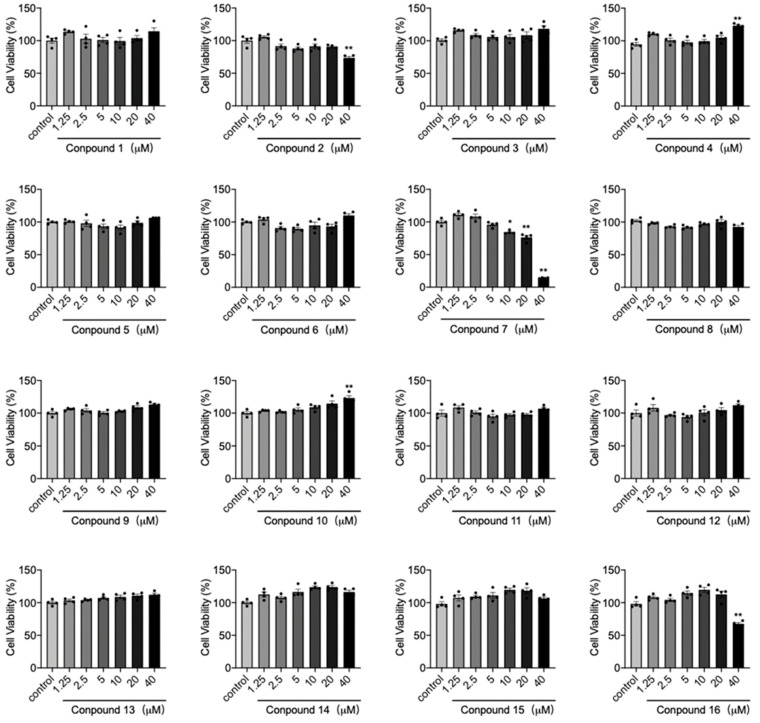
Raw264.7 cells were treated with compounds **1**–**16** at concentrations of 1.25, 2.5, 5, 10, 20, and 40 *μ*M for 24 h. Cell viability was assessed using a cytotoxicity assay with CCK-8 reagent. * *p* < 0.05, ** *p* < 0.01 vs. control group, n = 4.

Compounds **1**–**16** were assessed for their anti-inflammatory activity on the expression of pro-inflammatory cytokine factors *Tnfα* and *Ifnb1* in LPS- or DMXAA-induced Raw264.7 cells. Prior to exploring their anti-inflammatory properties, the potential toxicity of these compounds in Raw264.7 cells was evaluated to ensure that their effects were not confounded with cytotoxicity. The results demonstrated that most compounds had no significant impact on the viability of Raw264.7 cells, except for compounds **2**, **7**, and **16**, which exhibited some reduction in cell viability at high concentrations up to 40 *μ*M (Figure 4).

Cell apoptosis in macrophages is intricately associated with macrophage polarization, allowing macrophages to alter their phenotype and carry out diverse functions in response to changes in the microenvironment. The two main polarization states are classically activated macrophages (M1) and alternatively activated macrophages (M2). M1 macrophages exhibit characteristics such as pro-inflammatory mediator production, promotion of cell cytotoxicity, and antimicrobial abilities. Conversely, M2 macrophages possess anti-inflammatory properties, participate in tissue repair, regulate the immune response, and demonstrate anti-inflammatory capabilities. M1 macrophages induce cell death by releasing toxic molecules such as toxins, oxidants, and proteases, thereby augmenting their antimicrobial and antitumor effects. Nonetheless, excessive activation of M1 macrophages can result in tissue damage and inflammatory responses [44]. However, excessive activation of M1 macrophages can lead to tissue damage and inflammatory responses. The experimental findings revealed that compound **7** exerted a more pronounced inhibitory effect on the survival of Raw264.7 cells at a concentration of 40 µM compared to other compounds. This suggests that compound **7** may promote the polarization of M1 macrophages while inhibiting the expression of growth-related proteins such as P38, Akt, and Wnt and promoting the expression of apoptosis-related genes such as Bcl-2, Bcl-xL, and Mcl-1. Consequently, this inhibits macrophage proliferation and promotes macrophage apoptosis. Moreover, the inhibitory effect of compound **7** on the viability of Raw264.7 cells at a concentration of 40 µM also suggests its potential to inhibit the proliferation and differentiation of tumor cells.

**Figure 5:**
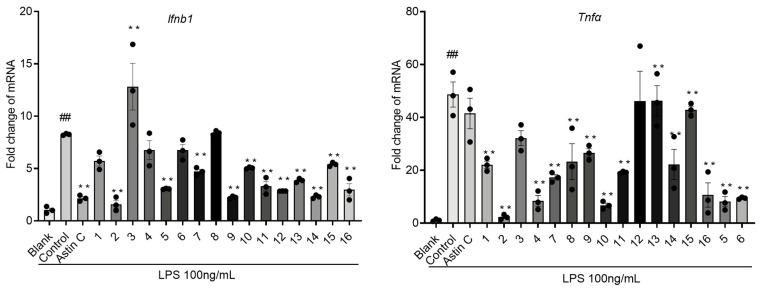
Raw264.7 cells were pretreated with compounds **1**–**16** at a concentration of 20 *μ*M for 2 h. Subsequently, the cells were stimulated with LPS (100 ng/mL) for 6 h. Total RNA was then extracted from the cells and subjected to a quantitative real-time polymerase chain reaction (*q*RT-PCR) to analyze the expression of a panel of genes associated with the innate immune response. ** *p* < 0.01 vs. Control group; ## *p* < 0.01 vs. Blank group, n = 3.

The expression of inflammatory factors in Raw264.7 cells, such as *Tnfα* and *Ifnb1*, which were stimulated by LPS, was suppressed by the administration of compounds **1**–**16** (Figure 5).

**Figure 6:**
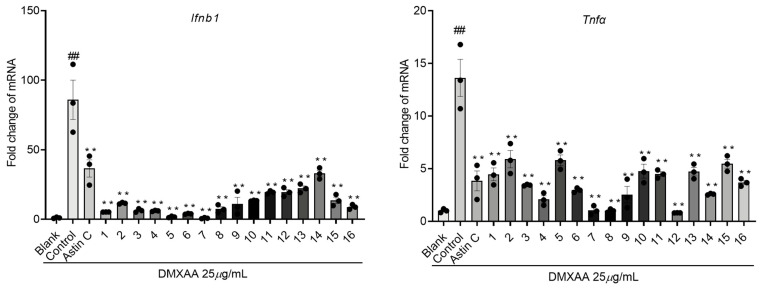
Raw264.7 cells were preincubated with compounds **1**–**16** (20 *μ*M) and Astin C (20 nM) for a duration of 2 h, followed by stimulation with DMXAA (25 *μ*g/mL) for 6 h. Total RNA was extracted from the cells and subjected to *q*RT-PCR analysis to evaluate the expression of a panel of genes associated with innate immune-responsive genes. ** *p* < 0.01 vs. Control group; ## *p* < 0.01 vs. Blank group, n = 3.

The above results demonstrate that the compounds exhibit good inhibitory activity against type I interferon, and the cGAS-STING pathway in the innate immune response is an important mechanism for regulating type I interferon responses [45,46,47]. The expression levels of *Tnfα* and *Ifnb1* in the STING pathway have practical importance in immune regulation, inflammatory diseases, and therapeutic interventions, serving as key indicators of immune activation, effectiveness against pathogens, and the extent of inflammation [48,49]. Therefore, we further used the specific activator DMXAA of the cGAS-STING pathway to establish an activation model and evaluate the activity of these compounds. Using astin C (20 nM) as a positive control and a natural inhibitor of the STING pathway [50], the results indicate that all compounds significantly inhibit the expression levels of *Tnfα* and *Ifnb1* (Figure 6). In the stimulation of Raw264.7 cells with LPS, compounds **2**, **9**, and **14** showed significant inhibition of the inflammatory cytokine *Ifnb1* expression, while compounds **2**, **4**, and **5** exhibited strong inhibition of the inflammatory cytokine *Tnfα* expression. When Raw264.7 cells were stimulated with DMXAA, compounds **1**, **5**, and **7** effectively suppressed the inflammatory cytokine *Ifnb1* expression, whereas compounds **7**, **8**, and **11** demonstrated the best inhibition of the inflammatory cytokine *Tnfα* expression. Notably, newly isolated compounds **2** and **11** demonstrated potent anti-inflammatory activity. Hence, it is probable that compounds **1**–**16** exert their anti-inflammatory effects through the modulation of the cGAS-STING pathway.

## 3. Materials and Methods

### 3.1. General Experimental Procedures

Optical rotations were determined with a Autopol VI polarimeter (Rudolph, Wood County, WI, USA). UV spectra and ECD spectra were taken in MeOH on a Jasco-715 spectropolarimeter. Infrared spectra were recorded on a Nicolet iN10 (micro) spectrometer (Thermo Fisher Scientific, Waltham, MA, USA). The NMR spectra were recorded on a Bruker DRX-600 (Bruker, Germany) spectrometer with chemical shifts reported relative to the residual CDCl_3_ (*δ*_H_ 7.26 ppm, *δ*_C_ 77.0 ppm) and CD_3_OD (*δ*_H_ 3.31 ppm; *δ*_C_ 49.0 ppm). The HRESIMS analyses were performed on a Q Exactive Plus Orbitrap (Thermo Fisher Scientific, Waltham, MA, USA) mass spectrometer. The UPLC-MS analysis of total ion chromatogram (TIC) was obtained using Agilent 1290 Infinity-6538 UHD (Agilent, Santa Clara, CA, USA) and Accurate-Mass QTOF/MS (Agilent, Santa Clara, CA, USA). Semipreparative HPLC was performed on a Waters 1525 (Waters, Milford, MA, USA) with a YMC Pack ODS-A column (250 × 10 mm, 5 *μ*M; YMC, Kyoto, Japan). Sephadex LH-20 gel (GE Healthcare Bio-Sciences AB, Uppsala, Sweden) and silica gel (200–300 mesh, 300–400 mesh; Yantai Chemical Engineering Institure, Yantai, China) were used for column chromatography. Precoated silica gel plates (HSGF-254; Yantai Chemical Engineering Institure, Yantai, China) were used for thin-layer chromatography (TLC). Spots were detected on TLC under UV or by heating after spraying with an anisaldehyde sulfuric acid reagent.

### 3.2. Fungal Material

*P. oxalicum* HL-44 was isolated from the soft coral *S*. *gaweli* that was collected from the Xisha area of the South China Sea at a depth of 15 m in Aug 2018 and identified as *P. oxalicum* using 18sRNA sequence (GenBank accession number MG585101.1). A voucher strain of this fungus (internal strain No. HL-44) was deposited at Tongji University, Shanghai, China.

### 3.3. Screening Culture Medium and Cultivation

Five media, including Czapek Dox Agar (CZA) medium, Glucose Peptone Yeast (GPY) medium, Potato Dextrose Agar (PDA) medium, Rose Bengal Medium (RBM), and Rice medium, were used in the fermentation to compare the chemical diversity of fungal HL-44. UPLC-MS analysis of five extracts indicated that the PDA medium allowed the strain to produce metabolites with large molecular weights and more chemical diversity (*m*/*z* range from 408 to 688, *t_R_* from 10.5 to 14.5 min, Figure S2). The PDA medium was then selected for scale-up fermentation.

### 3.4. Extraction and Isolation

The culture medium containing mycelia was cut into small pieces and extracted five times ultrasonically with EtOAc to afford 43.5 g of residue after removal of the solvent under reduced pressure. The crude extract was separated into ten fractions (Fr.1–10) with silica gel CC (80 mm × 150 mm, 810 g, 200–300 mesh), eluting with a gradient CH_2_Cl_2_/MeOH (100:0 *v*/*v* 5 L, 80:1 *v*/*v* 3 L, 60:1 *v*/*v* 5 L, 40:1 *v*/*v* 3 L, 20:1 *v*/*v* 5 L, 5:1 *v*/*v* 5 L). Fr. 1 (3.3 g) was subjected to a Sephadex LH-20 CC (4 cm × 120 cm) using CH_2_Cl_2_/MeOH (*v*/*v* 2:1) as eluent to obtain eight subfractions (Fr.1a-h). Fr.1c (411.6 mg) was separated with silica gel CC (10 mm × 150 mm, 14 g, 300–400 mesh) using gradient petroleum (PE) in Me_2_CO (39:1 *v*/*v* 40 mL, 24:1 *v*/*v* 50 mL, 19:1 *v*/*v* 40 mL, 1:1 *v*/*v* 20 mL), then split with HPLC to yield compound **1** (0.9 mg, MeOH/H_2_O 98:2, 2 mL/min, *t_R_* 64 min). Fr.1e (112.8 mg) was separated with silica gel CC (15 mm × 200 mm, 38 g, 300–400 mesh) using PE/Me_2_CO (39:1 *v*/*v* 120 mL, 29:1 *v*/*v* 120 mL, 19:1 *v*/*v* 160 mL, 9:1 *v*/*v* 100 mL, 4:1 *v*/*v* 50 mL, 2:1 *v*/*v* 30 mL), then split with HPLC to yield compound **11** (1.6 mg, MeOH/H_2_O 90:10, 2 mL/min, *t_R_* 51 min) and compound **6** (12.8 mg, MeOH/H_2_O 87:13, 2 mL/min, *t_R_* 55 min). Fr.1f (788.1 mg) was separated with silica gel CC (20 mm × 150 mm, 55 g, 300–400 mesh) using PE/Me_2_CO (39:1 *v*/*v* 100 mL, 19:1 *v*/*v* 100 mL, 14:1 *v*/*v* 120 mL, 9:1 *v*/*v* 100 mL, 1:1 *v*/*v* 50 mL), then split with HPLC to yield compound **14** (1.5 mg, MeOH/H_2_O 88:12, 2 mL/min, *t_R_* 74 min), compound **12** (0.6 mg, MeOH/H_2_O 88:12, 2 mL/min, *t_R_* 81 min) and compound **15** (8.4 mg, MeOH/H_2_O 88:12, 2 mL/min, *t_R_* 95 min). Fr. 2 (794.5 mg) was fractionated using Sephadex LH-20 CC (CH_2_Cl_2_/MeOH *v*/*v* 2:1, 4 cm × 120 cm) and then purified with silica gel CC (15 mm × 150 mm, 30 g, 300–400 mesh) using a gradient PE in Me_2_CO (39:1 *v*/*v* 80 mL, 19:1 *v*/*v* 100 mL, 9:1 *v*/*v* 100 mL, 1:1 *v*/*v* 40 mL), and HPLC to afford compound **4** (2.8 mg, MeOH/H_2_O 97:3, 2 mL/min, *t_R_* 52 min) and compound **7** (4.4 mg, MeOH/H_2_O 95:5, 2 mL/min, *t_R_* 41 min). Fr. 4 (2.2 g) was fractionated using Sephadex LH-20 CC (CH_2_Cl_2_/MeOH *v*/*v* 2:1, 4 cm × 120 cm) and then purified with silica gel CC (20 mm × 150 mm, 55 g, 300–400 mesh) using a gradient PE in Me_2_CO (29:1 *v*/*v* 90 mL, 19:1 *v*/*v* 100 mL, 14:1 *v*/*v* 90 mL, 9:1 *v*/*v* 100 mL, 4:1 *v*/*v* 50 mL) and HPLC to give compound **5** (4.4 mg, MeOH/H_2_O 80:20, 2 mL/min, *t_R_* 65 min), compound **3** (0.5 mg, CH_3_CN/H_2_O 70:30, 2 mL/min, *t_R_* 185 min), and compound **16** (1.9 mg, MeOH/H_2_O 80:20, 2 mL/min, *t_R_* 73 min). Fr. 5 (755.9 mg) was fractionated using Sephadex LH-20 CC (CH_2_Cl_2_/MeOH *v*/*v* 2:1, 4 cm × 120 cm) and then purified with silica gel CC (15 mm × 150 mm, 30 g, 300–400 mesh) using a gradient PE in Me_2_CO (29:1 *v*/*v* 90 mL, 14:1 *v*/*v* 90 mL, 9:1 *v*/*v* 120 mL, 4:1 *v*/*v* 50 mL) and HPLC to give compound **10** (1.8 mg, MeOH/H_2_O 88:12, 2 mL/min, *t_R_* 69 min). Fr. 7 (740.6 mg) was fractionated using Sephadex LH-20 CC (CH_2_Cl_2_/MeOH *v*/*v* 2:1, 4 cm × 120 cm) and then purified with silica gel CC (15 mm × 150 mm, 30 g, 300–400 mesh) using PE/Me_2_CO (8:1 *v*/*v* 90 mL and 4:1 *v*/*v* 50 mL) to give compound **13** (2.0 mg). Fr. 9 (2.4 g) was fractionated using Sephadex LH-20 CC (CH_2_Cl_2_/MeOH *v*/*v* 2:1, 4 cm × 120 cm) and then purified with silica gel CC (10 mm × 150 mm, 14 g, 300–400 mesh) using PE/ Me_2_CO (8:1 *v*/*v* 90 mL, 6:1 *v*/*v* 70 mL, 4:1 *v*/*v* 30 mL) and HPLC to give compound **2** (1.3 mg, MeOH/H_2_O 87:13, 2 mL/min, *t_R_* 27 min). Fr. 10 (1.7 g) was fractionated using Sephadex LH-20 CC (CH_2_Cl_2_/MeOH *v*/*v* 2:1, 4 cm × 120 cm) and then purified with silica gel CC (15 mm × 150 mm, 30 g, 300–400 mesh) using PE/Me_2_CO (19:1 *v*/*v* 80 mL, 9:1 *v*/*v* 120 mL, 7:1 *v*/*v* 80 mL, 5:1 *v*/*v* 120 mL, 2:1 *v*/*v* 90 mL) and HPLC to give compound **9** (1.4 mg, MeOH/H_2_O 90:10, 2 mL/min, *t_R_* 35 min) and compound **8** (0.9 mg, MeOH/H_2_O 90:10, 2 mL/min, *t_R_* 47 min).

Characterization data of compound **1**: (22*E*,24*S*)-9*α*,15*α*-dihydroxyergosta-4,6,8(14),22-tetraen-3-one 15-palmitate (**1**): yellowish oil, ${\left[\alpha \right]}_{D}^{25}$ +43.40 (*c* 0.10, CH_3_OH); UV (CH*_3_*OH) *λ*_max_ (log *ε*) 330 (2.49), 265 (2.02) nm; ECD (CH_3_OH, *c* 1.5 × 10^−4^) *λ*_max_ (∆ *ε*) 241 (+1.80), 320 (-1.43), 361 (+2.76) nm; IR (micro) *ν*_max_ 3443, 2929, 2854, 1733, 1664, 1595, 1460, 1260, 1094, 1033, 970, 801 cm^−1^; ^1^H and ^13^C NMR data see Table 1; HRESIMS *m*/*z* 663.53448 [M + H]^+^ (calcd for C_44_H_71_O_4_, 663.53469).

### 3.5. Cell Viability Detection

The viability of Raw264.7 cells was evaluated using the CCK-8 assay. Initially, the cells were seeded at a density of 3 × 10^4^ cells/well in 96-well plates and allowed to incubate overnight. The cells were treated with the tested compounds at concentrations ranging from 1.25 to 40 *μ*M for 24 h. After removing the culture medium, a CCK-8 solution diluted in Dulbecco’s modified Eagle medium was added to each well. Following a 1-h incubation, the absorbance was measured at 450 nm using a multifunctional microplate spectrophotometer. Cell viability was then calculated as a percentage relative to the blank group [51].

### 3.6. Quantitative Real-Time PCR (qPCR)

Raw264.7 cells were pretreated with tested compounds at a concentration of 20 *μ*M, along with astin C [50] at a concentration of 20 nM, for 2 h. Subsequently, the cells were stimulated with LPS at a concentration of 100 ng/mL and DMXAA at a concentration of 25 *μ*g/mL for 6 h [52]. Total RNA was extracted from Raw264.7 cells using a triazole reagent supplied by Thermo Fisher Scientific. cDNA synthesis was performed using SuperScript III reverse transcriptase obtained from Invitrogen. Real-time PCR analysis was conducted using a PrimeScript RT reagent kit from Takara. The relative expression levels of the target genes were quantitatively normalized to the expression level of Gapdh using the ΔΔct method. Primer sequences are *Ifnb1*, forward 5′-GCACTGGGTGGAATGAGACT-3′ and reverse 5′-AGTGGAGAGCAGTTGAGGACA-3′; *Tnfα*, forward 5′-GTCCCCAAAGGGATGAGAAGTT-3′ and reverse 5′-GTTTGCTACGACGTGGGCTACA-3′ [52].

### 3.7. Statistical Analysis

The statistical analyses were conducted using GraphPad Prism 8.0. A one-way analysis of variance (ANOVA) was applied to the data, followed by Tukey’s test for comparisons against the blank and control groups. A significance level of *p* < 0.05 was considered statistically significant.

## 4. Conclusions

With chemical analysis of the PDA medium extract of the fungus *P. oxalicum* HL-44 associated with the soft coral *S. gaweli*, a new ergostane-type sterol ester (**1**) was isolated together with fifteen derivatives (**2**–**16**). Their structures were determined using spectroscopic analyses and comparisons with reported data. This is the first report of compounds **2** and **11** from the family of Eurotiaceae. The isolation of ergosteroid derivatives with varying degrees of oxidation revealed a remarkable range of chemical diversity, expanding the ergosteroid family associated with this fungus. In an in vitro biotest, these compounds demonstrated potent anti-inflammatory activities at a concentration of 20 *μ*M, effectively suppressing the expression of inflammatory factors such as *Tnfα* and *Ifnb1* in Raw264.7 cells induced with LPS or DMXAA. Among DMXAA-induced Raw264.7 cells, compounds **7** and **8** exhibited the highest level of inhibition in terms of *Tnfα* and *Ifnb1* expression. Conversely, in LPS-induced Raw264.7 cells, compounds **2** and **4** displayed the most pronounced inhibitory effects on *Tnfα* and *Ifnb1* expression. This study provides valuable insight into the chemical diversity of ergosteroid derivatives and their potential as anti-inflammatory agents.

## Figures and Tables

**Table 1:** ^1^H and ^13^C NMR spectral data of compound **1** in CDCl_3._ (500 MHz for ^1^H and 125 MHz for ^13^C, *δ* in ppm, *J* in Hz).

Position	*δ*_C_	*δ*_H_ (*J* in Hz)	Position	*δ*_C_	*δ*_H_ (*J* in Hz)
1	27.5	2.53 m, 1.80 m	19	21.0	1.14 s
2	33.9	2.53 m	20	38.4	2.12 m
3	199.3	-	21	21.3	1.09 d (6.6)
4	127.3	5.91 s	22	134.4	5.19 dd (15.3, 8.2)
5	160.4	-	23	133.5	5.26 dd (15.3, 7.6)
6	126.5	6.13 d (9.8)	24	43.1	1.85 m
7	129.9	6.41 d (9.8)	25	33.1	1.47 m
8	132.0	-	26	20.1	0.83 d (6.7)
9	72.8	-	27	19.7	0.81 d (6.8)
10	42.4	-	28	17.9	0.92 d (6.8)
11	25.6	2.02 m, 1.75 m	1′	173.6	-
12	32.1	2.00 m, 1.72 m	2′	34.7	2.30 t (7.2)
13	44.8	-	3′	25.2	1.61 m
14	154.0	-	4′–13′	29.8–29.3	1.30–1.23 m
15	71.8	5.81 d (7.1)	14′	32.0	1.25 m
16	37.6	1.94 m, 1.72 m	15′	22.8	1.31 m, 1.24 m
17	53.1	1.63 m	16′	14.2	0.88, t (6.6)
18	19.3	0.95 s			

s = singlet; d = doublet; t = triplet; m = multiplet; dd = doublet of doublet.

## Data Availability

All data are available in the main text or the Supplementary Materials.

## Supplementary Materials

The following supporting information can be downloaded at https://www.mdpi.com/article/10.3390/molecules28237784/s1. Figure S1. HL-44 cultured on PDA medium (A), CZA medium (B), GPY medium (C), RBM, (D) Rice medium, and (E); Figure S2. Total Ion Chromatography (TIC) of 5 candidate media extracts: PDA medium (A), CZA medium (B), GPY medium (C), RBM (D), and Rice medium (E); Figures S3–S13. 1H-NMR, 13C-NMR, DEPT, 1H-1H COSY, HSQC, HMBC, NOESY, UV, IR, HRESIMS, and CD spectra of compound 1; Figure S14. OR data of compound 1 in CH3OH; Figure S15. The 1H-NMR spectrum of previously reported compounds **2**–**16**.
