# Supplementary material for: Anti-Inflammatory Ergosteroid Derivatives from the Coral-Associated Fungi Penicillium oxalicum HL-44
Source: Molecules. 2023 Nov 26;28(23):7784. doi: 10.3390/molecules28237784 (PMC10708211; doi:10.3390/molecules28237784)
Supplement: Supplementary file 1 [file molecules-28-07784-s001.zip › molecules-2595041-supplementary.pdf]

Anti-inflammatory ergosteroid derivatives from the coral-associated fungi *Penicillium oxalicum* HL-44

Cheng Pang [1,2,†], Yu-Hong Chen [3,†], Hui-Hui Bian [3], Jie-Ping Zhang [2], Li Su, Hua Han [2,*] and Wen Zhang [1,2,*]

1 School of Pharmaceutical Sciences, Zhejiang Chinese Medical University, Gao-Ke Rd., Hangzhou 311402, China

2 School of Medicine, Tongji University, 1238 Gonghexin Rd., Shanghai 200070, China

3 Institute of Translational Medicine, Shanghai University, 99 Shangda Rd., Shanghai 200444, China

* Correspondence: wenzhang1968@163.com; hanhua@tongji.edu.cn

† These authors contributed equally to this work.

Abstract: To obtain the optimal fermentation condition for more abundant secondary metabolites, Potato Dextrose Agar (PDA) medium was chosen for the scale-up fermentation of the fungus *Penicillium oxalicum* HL-44 associated with the soft coral *Sinularia gaweli*. The EtOAc extract of the fungi HL-44 was subjected to repeated column chromatography (CC) on silica gel and Sephadex LH-20 and semipreparative RP-HPLC to afford a new ergostane-type sterol ester (**1**) together with fifteen derivatives (**2**–**16**). Their structures were determined with spectroscopic analyses and comparisons with reported data. The anti-inflammatory activity of the tested isolates was assessed by evaluating the expression of pro-inflammatory factors *Tnfα* and *Ifnb1* in Raw264.7 cells stimulated with LPS or DMXAA. Compounds **2**, **9**, and **14** exhibited significant inhibition of *Ifnb1* expression, while compounds **2**, **4**, and **5** showed strong inhibition of *Tnfα* expression in LPS-stimulated cells. In DMXAA-stimulated cells, compounds **1**, **5**, and **7** effectively suppressed *Ifnb1* expression, whereas compounds **7**, **8**, and **11** demonstrated the most potent inhibition of *Tnfα* expression. These findings suggest that the tested compounds may exert their anti-inflammatory effects by modulating the cGAS-STING pathway. This study provides valuable insight into the chemical diversity of ergosteroid derivatives and their potential as anti-inflammatory agents.

Keywords: *Penicillium oxalicum*; coral-associated fungi; ergosteroid derivatives; anti-inflammatory activity

List of Figures S1-S15

Figures S1. HL-44 cultured on PDA medium (A); CZA medium (B); GPY medium (C); RBM (D) and Rice medium (E)
Figures S2. TIC of metabolites produced in five candidates of media: PDA medium (A) CZA medium (B); GPY medium (C); RBM (D) and Rice medium (E)
Figure S3. ^{1}H-NMR spectrum of compound **1** in $CDCl_3$
Figure S4. ^{13}C-NMR spectrum of compound **1** in $CDCl_3$
Figure S5. DEPT spectrum of compound **1** in $CDCl_3$
Figure S6. ^{1}H-^{1}H COSY spectrum of compound **1** in $CDCl_3$
Figure S7. HSQC spectrum of compound **1** in $CDCl_3$
Figure S8. HMBC spectrum of compound **1** in $CDCl_3$
Figure S9. NOESY spectrum of compound **1** in $CDCl_3$
Figure S10. UV spectrum of compound **1** in CH_3OH
Figure S11. IR spectrum of compound **1**
Figure S12. HRESIMS spectrum of compound **1**
Figure S13. CD spectrum of compound **1** in CH_3OH
Figure S14. OR data of compound **1** in CH_3OH
Figures S15. ^{1}H-NMR spectrum of previously reported compounds **2-16**

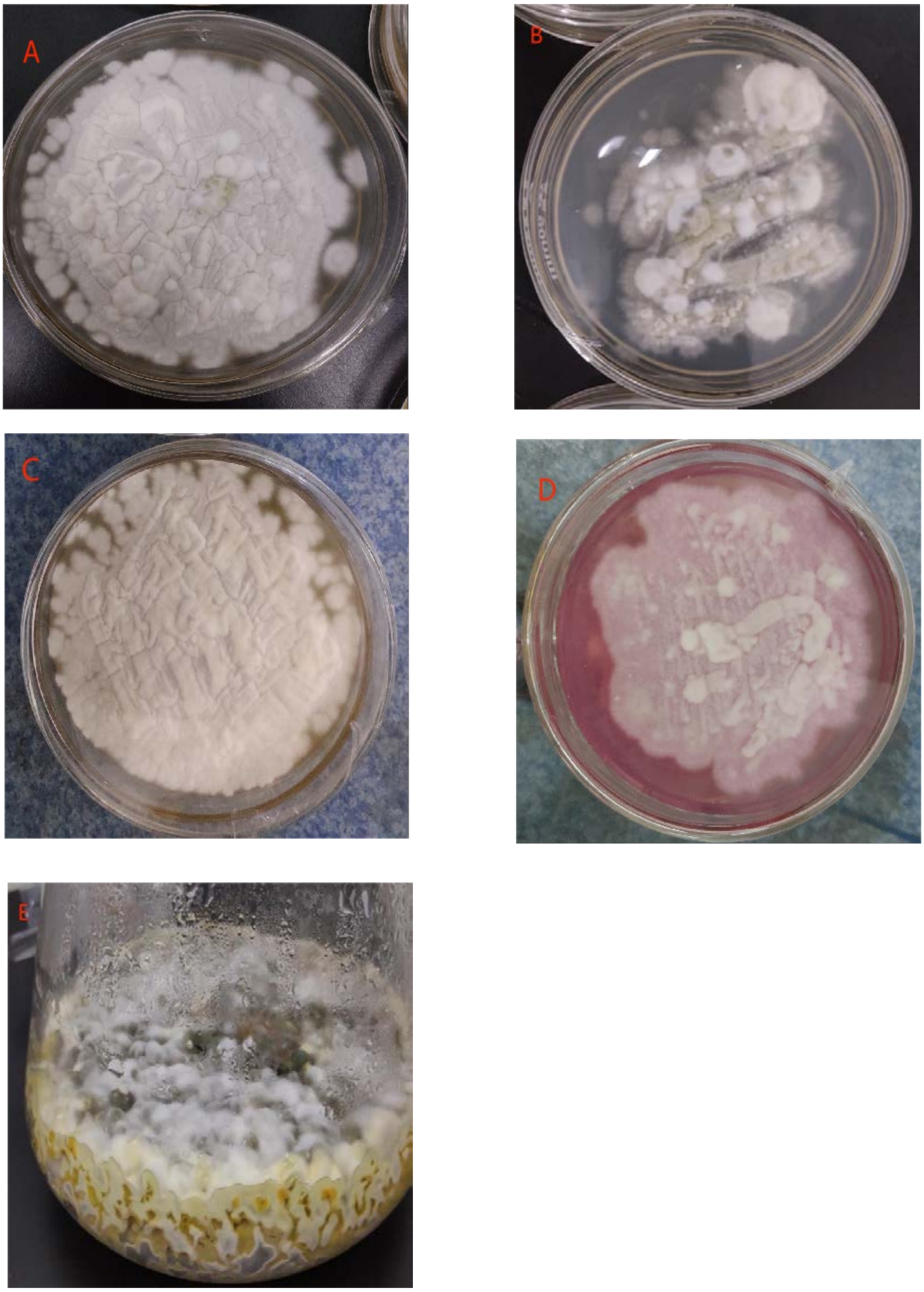

Figures S1. HL-44 cultured on PDA medium (A); CZA medium (B); GPY medium (C); RBM (D) and Rice medium (E)

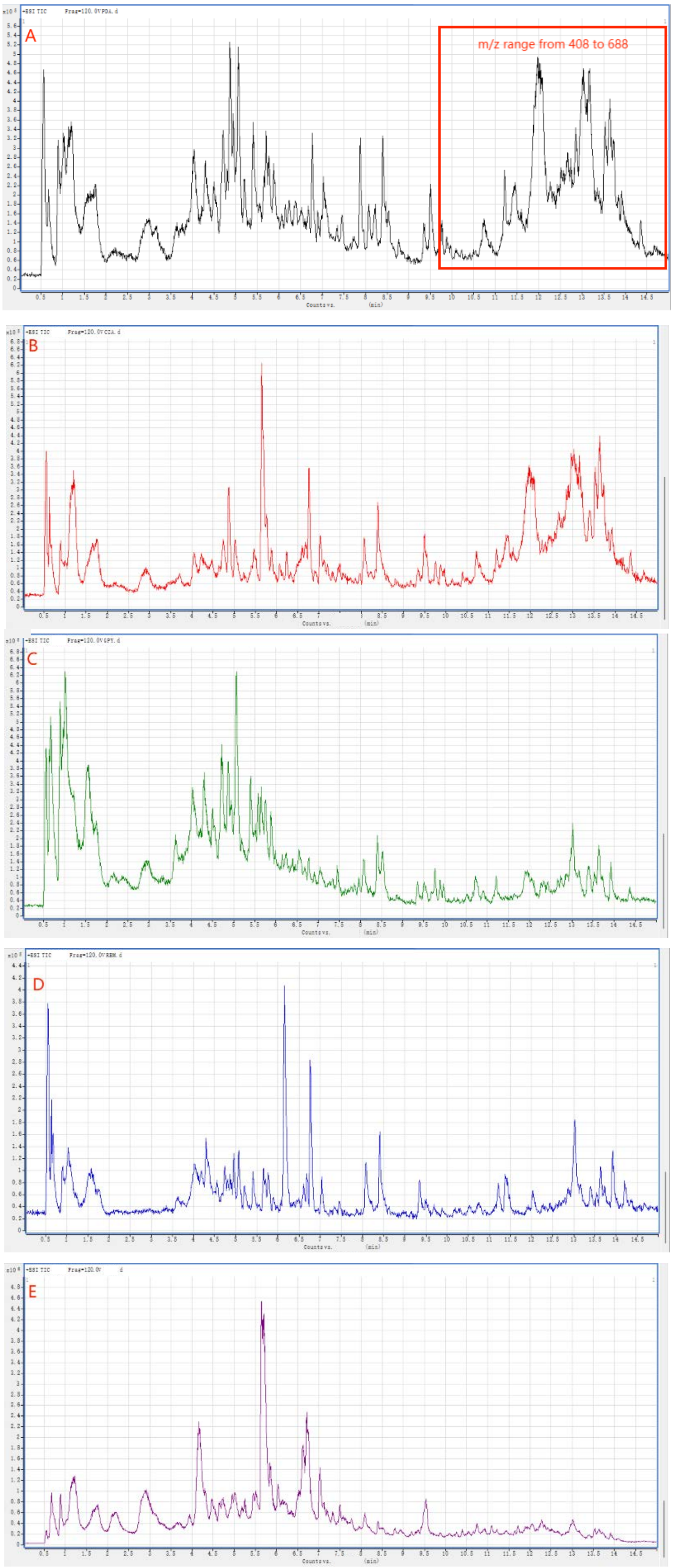

Figures S2. Total Ion Chromatography (TIC) of metabolites produced in five candidates of media: PDA medium (A) CZA medium (B); GPY medium (C); RBM (D) and Rice medium (E)

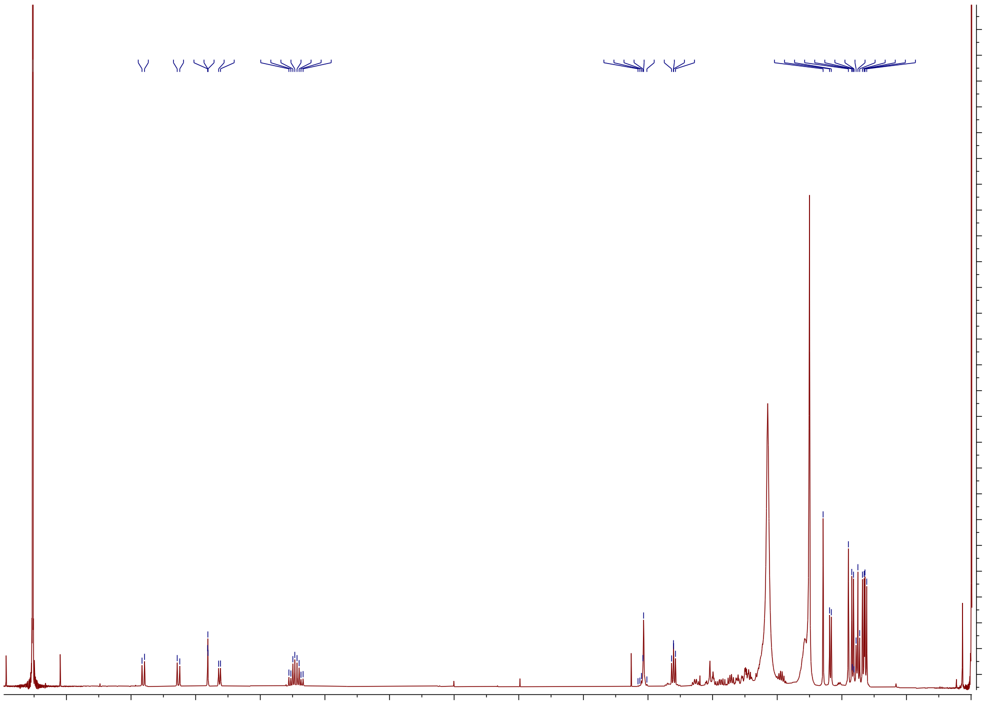

Figure S3. ^{1}H-NMR spectrum of compound **1** in $CDCl_3$

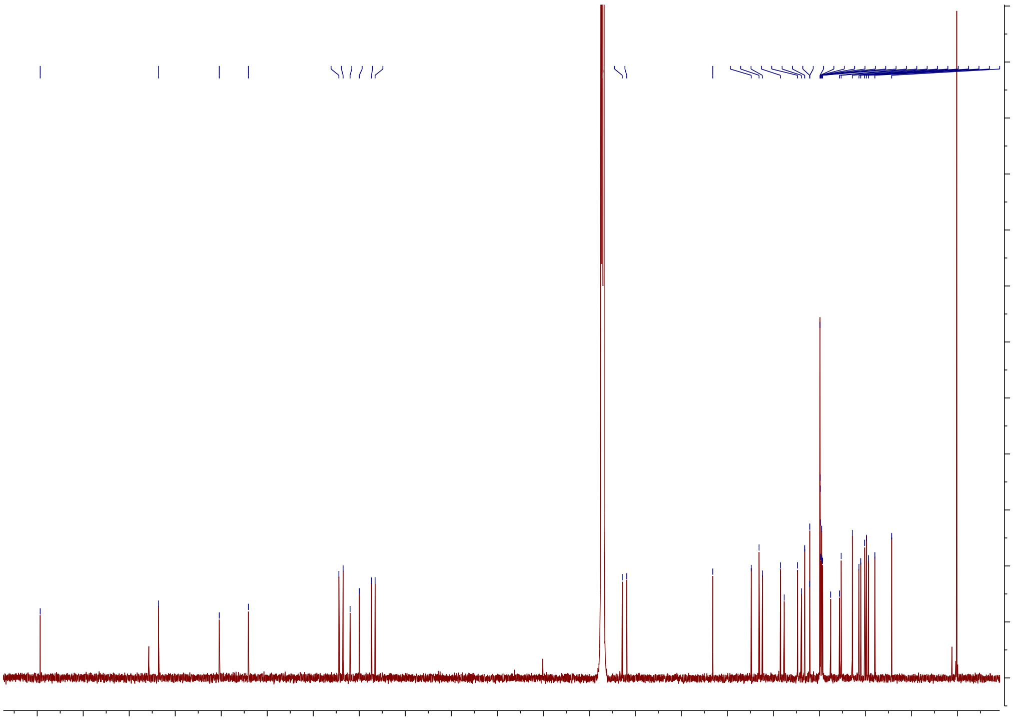

Figure S4. ^{13}C-NMR spectrum of compound **1** in $CDCl_3$

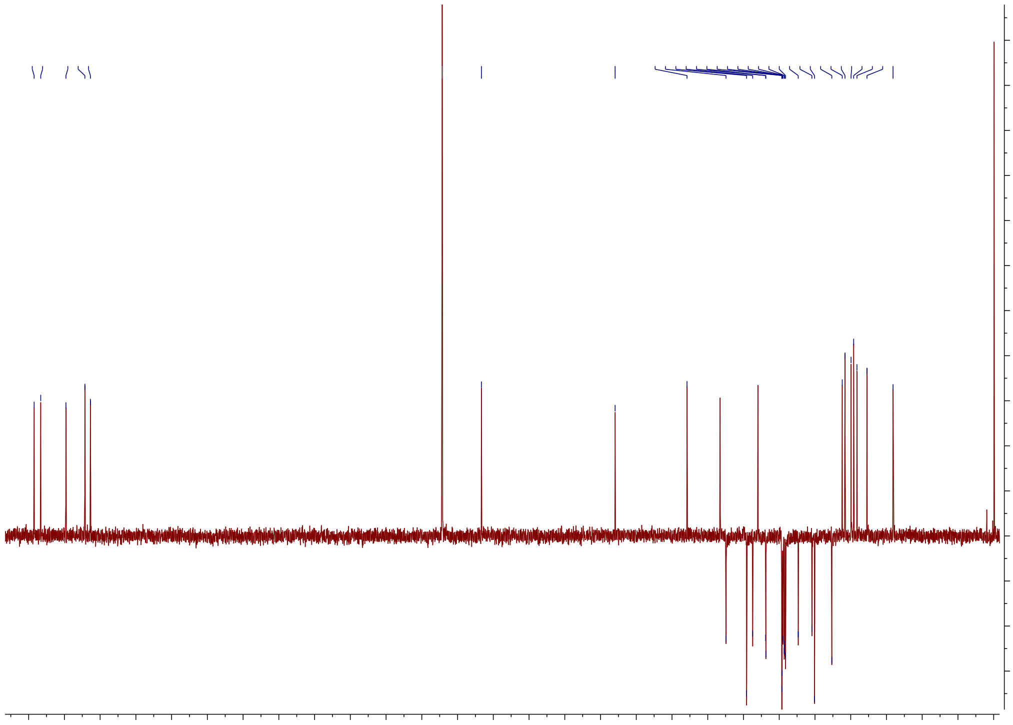

Figure S5. DEPT spectrum of compound **1** in $CDCl_3$

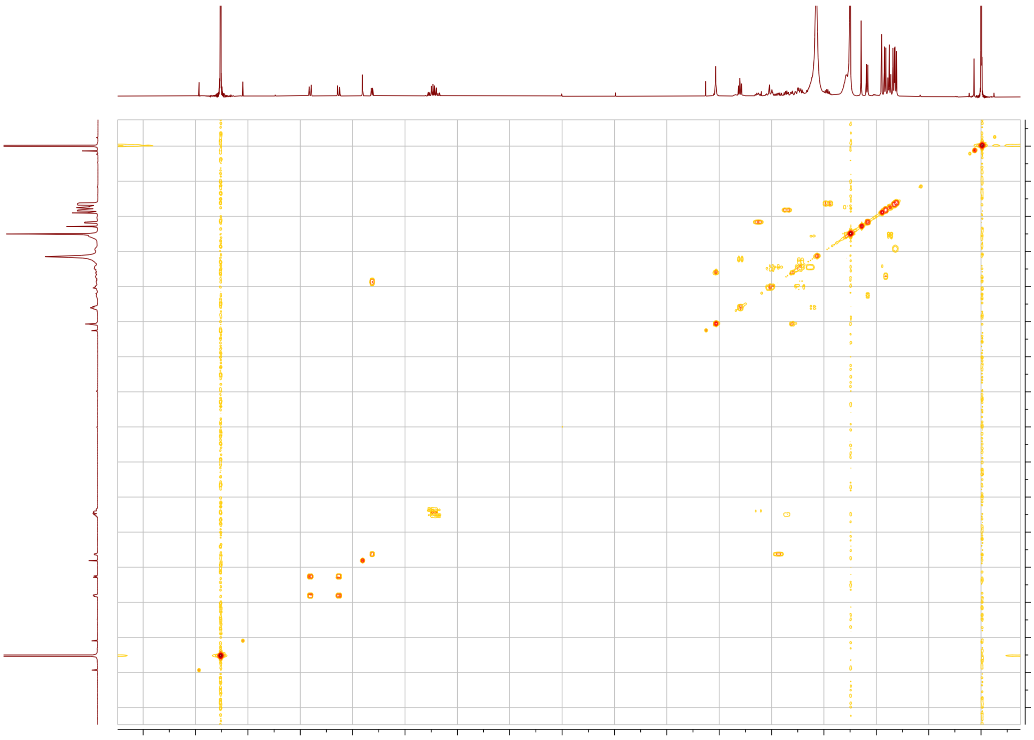

Figure S6. ^{1}H-^{1}H COSY spectrum of compound **1** in $CDCl_3$

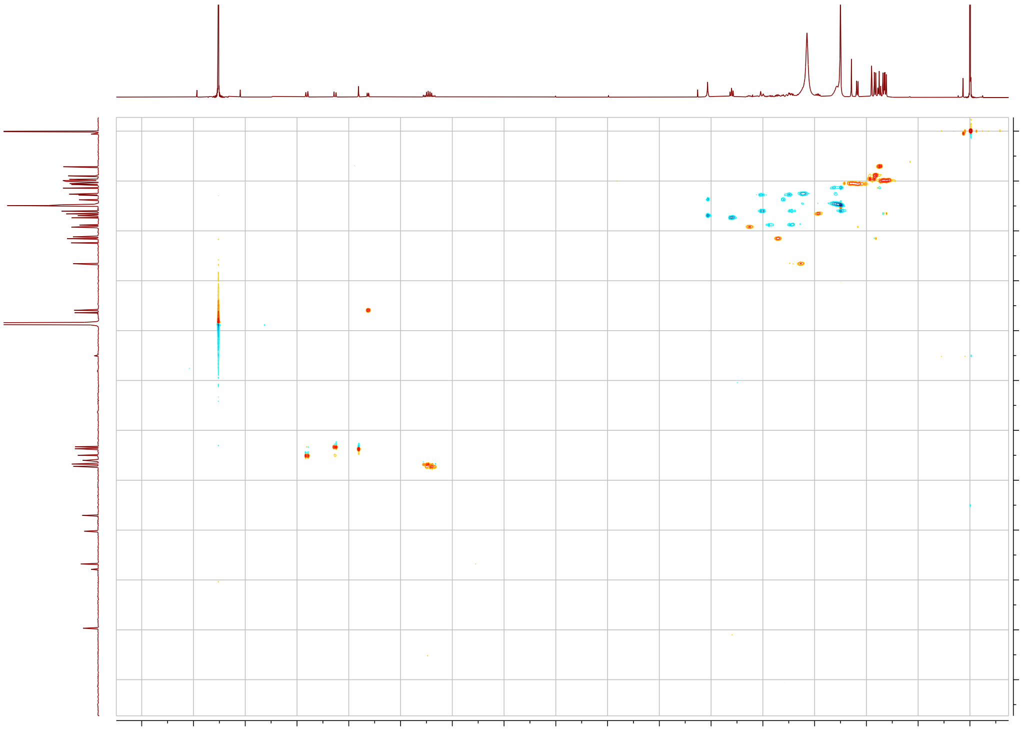

Figure S7. HSQC spectrum of compound **1** in $CDCl_3$

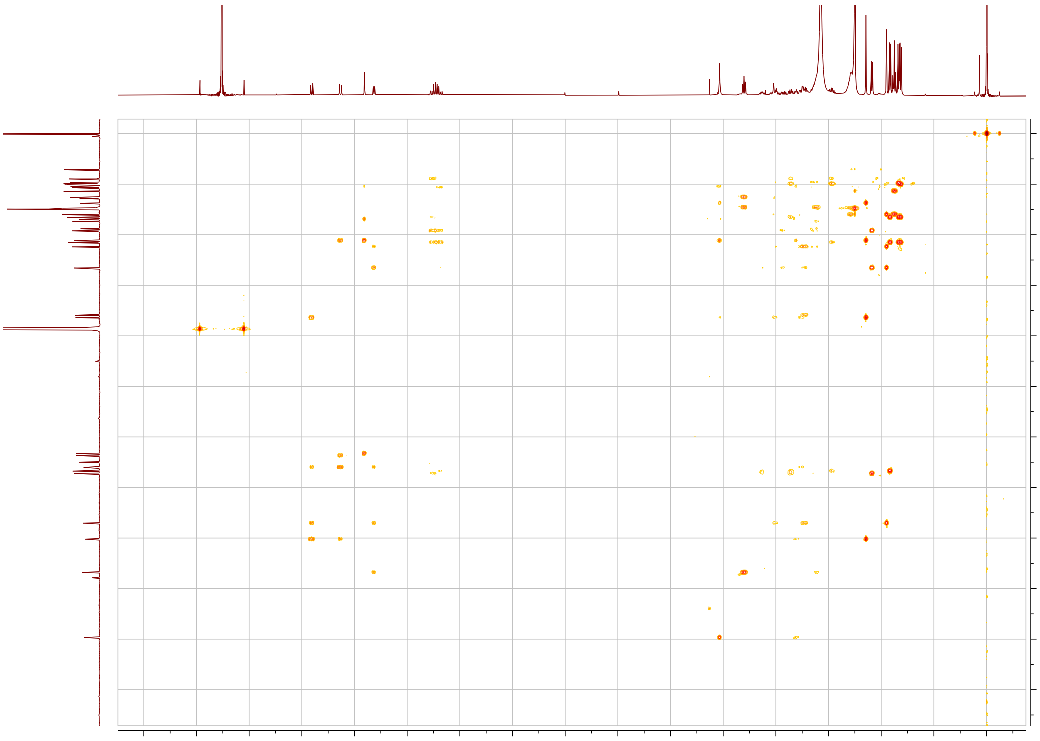

Figure S8. HMBC spectrum of compound **1** in $CDCl_3$

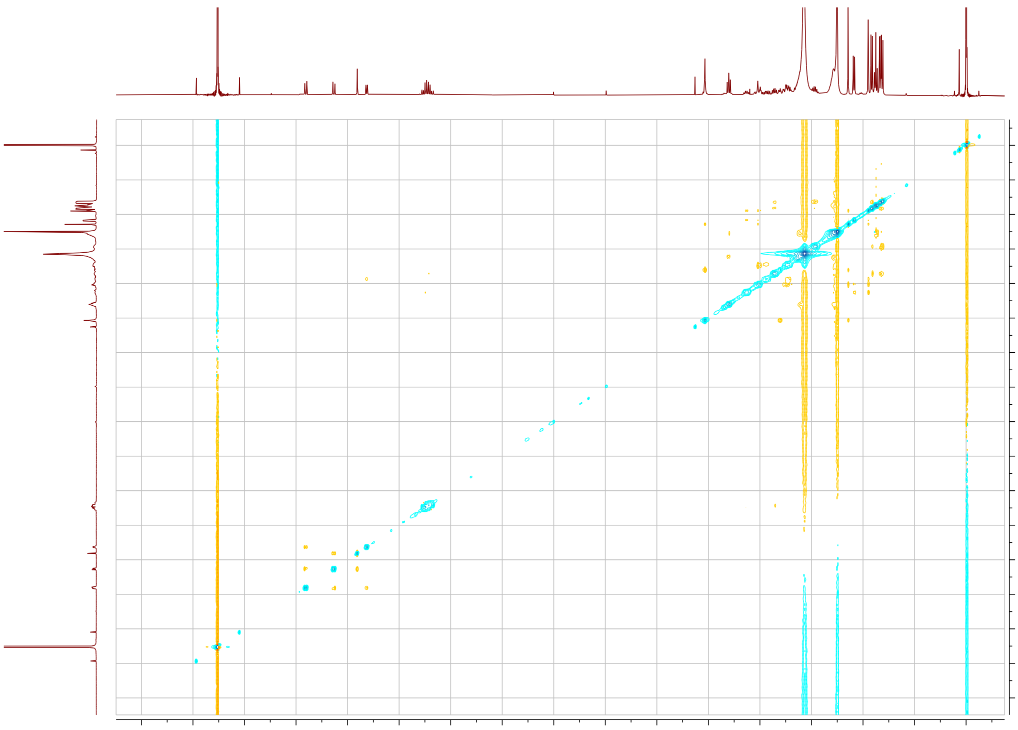

Figure S9. NOESY spectrum of compound **1** in $CDCl_3$

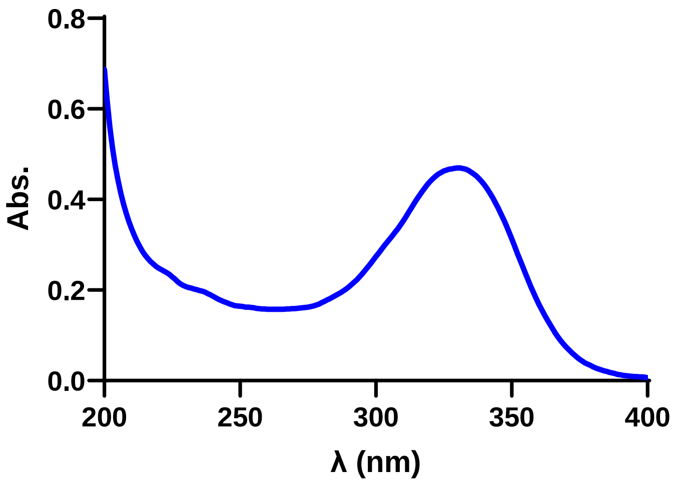

Figure S10. UV spectrum of compound **1** in CH_3OH
UV (CH_3OH) λ_{max} (log ε) 330 (2.49), 265 (2.02) nm

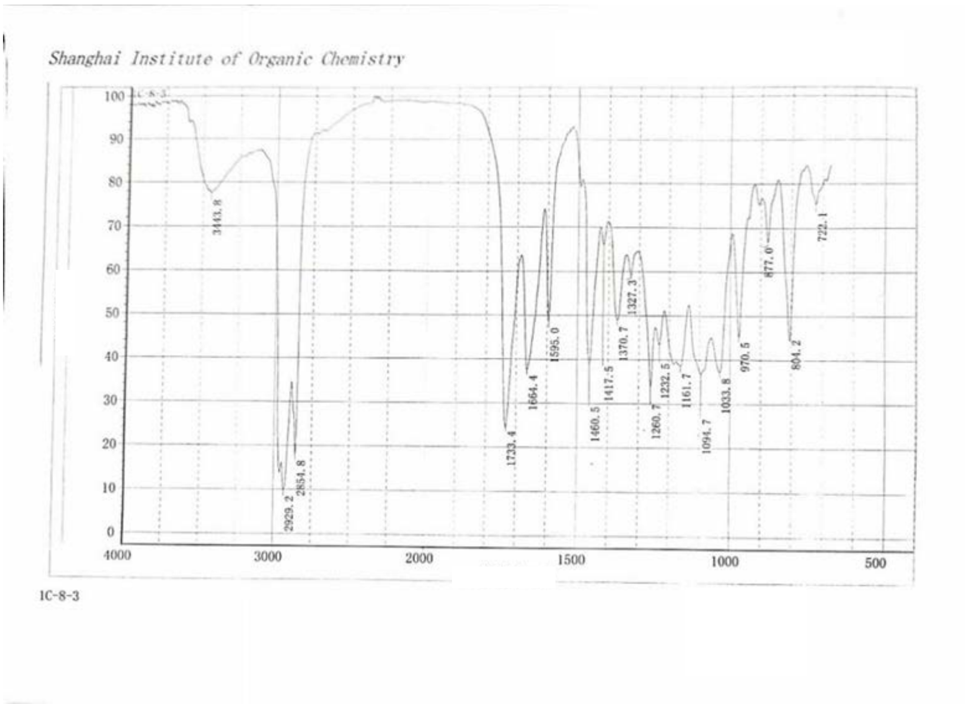

Figure S11. IR spectrum of compound **1**

Chromatogram and Results

Injection Details

Injection Name:	**Ff-1c-8-3**	*Run Time (min):*	**2.00**
Vial Number:	**RA8**	*Injection Volume:*	**1.00**
Injection Type:	**Unknown**	*Channel:*	**TIC**
Comment:	**20230313088**	*Wavelength:*	**n.a.**
Instrument Method:	**MS ESI+ 100-1500**	*Report Template:*	**HRMS Report**
Processing Method:	**MS Qual**	*Operator:*	**Liuju**
Injection Date/Time:	**15/Mar/23 15:41:18**	*Report Date/Time:*	**16/Mar/2023 09:54:29**

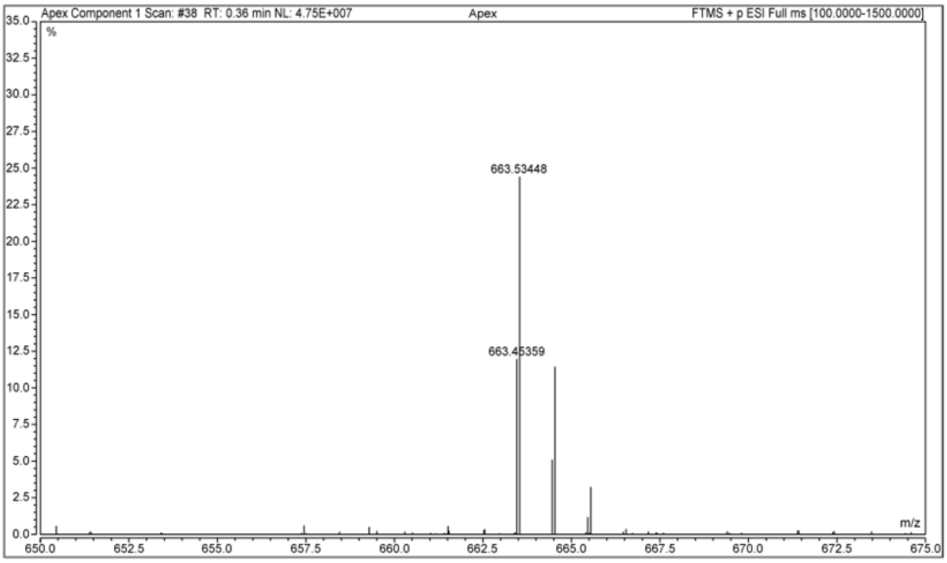

Mass spectrum Peak List

RT (min)	m/z	Theo. Mass	Charge	Delta (ppm)	Ion	Formula
0.36	663.53448	663.53469	1	-0.31	[M+H]+	C44H70O4

Figure S12. HRESIMS spectrum of compound **1**

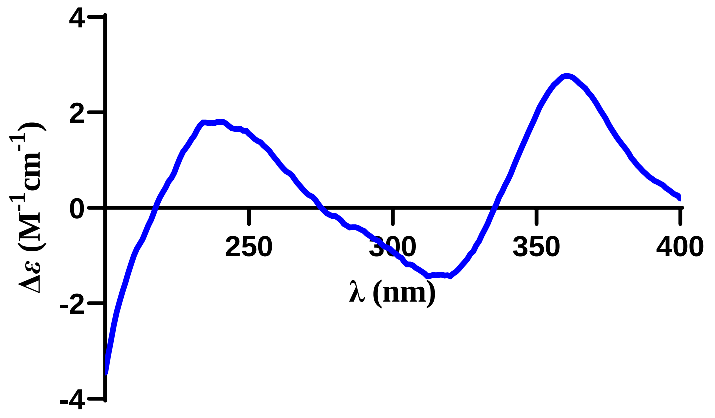

Figure S13. CD spectrum of compound **1** in CH_3OH

ECD (CH_3OH, *c* 1.5×10^{-4}) λ_{max} ($\Delta\varepsilon$) 241 (+1.80), 320 (-1.43), 361 (+2.76) nm

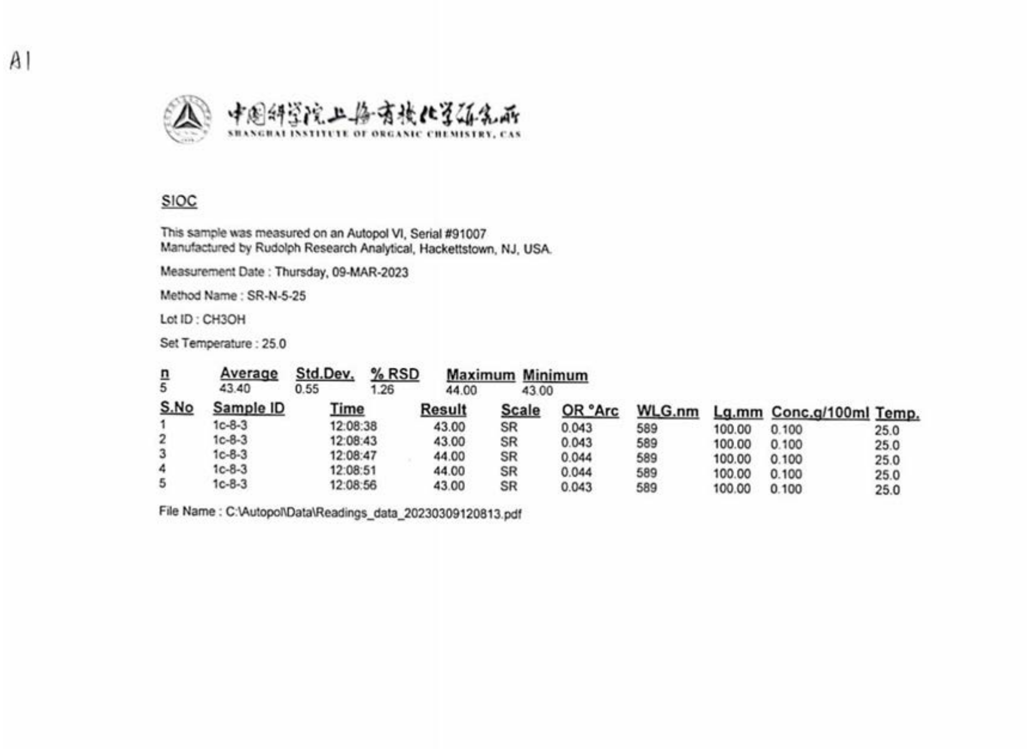

A1

中国科学院上海有机化学研究所
SHANGHAI INSTITUTE OF ORGANIC CHEMISTRY, CAS

SIOC

This sample was measured on an Autopol VI, Serial #91007
Manufactured by Rudolph Research Analytical, Hackettstown, NJ, USA.

Measurement Date : Thursday, 09-MAR-2023

Method Name : SR-N-5-25

Lot ID : CH3OH

Set Temperature : 25.0

n	Average	Std.Dev.	% RSD	Maximum	Minimum
5	43.40	0.55	1.26	44.00	43.00

S.No	Sample ID	Time	Result	Scale	OR °Arc	WLG.nm	Lg.mm	Conc.g/100ml	Temp.
1	1c-8-3	12:08:38	43.00	SR	0.043	589	100.00	0.100	25.0
2	1c-8-3	12:08:43	43.00	SR	0.043	589	100.00	0.100	25.0
3	1c-8-3	12:08:47	44.00	SR	0.044	589	100.00	0.100	25.0
4	1c-8-3	12:08:51	44.00	SR	0.044	589	100.00	0.100	25.0
5	1c-8-3	12:08:56	43.00	SR	0.043	589	100.00	0.100	25.0

File Name : C:\Autopol\Data\Readings_data_20230309120813.pdf

Figure S14. OR data of compound **1** in CH_3OH

Figures S15. ^{1}H-NMR spectrum of previously reported compounds **2-16**:

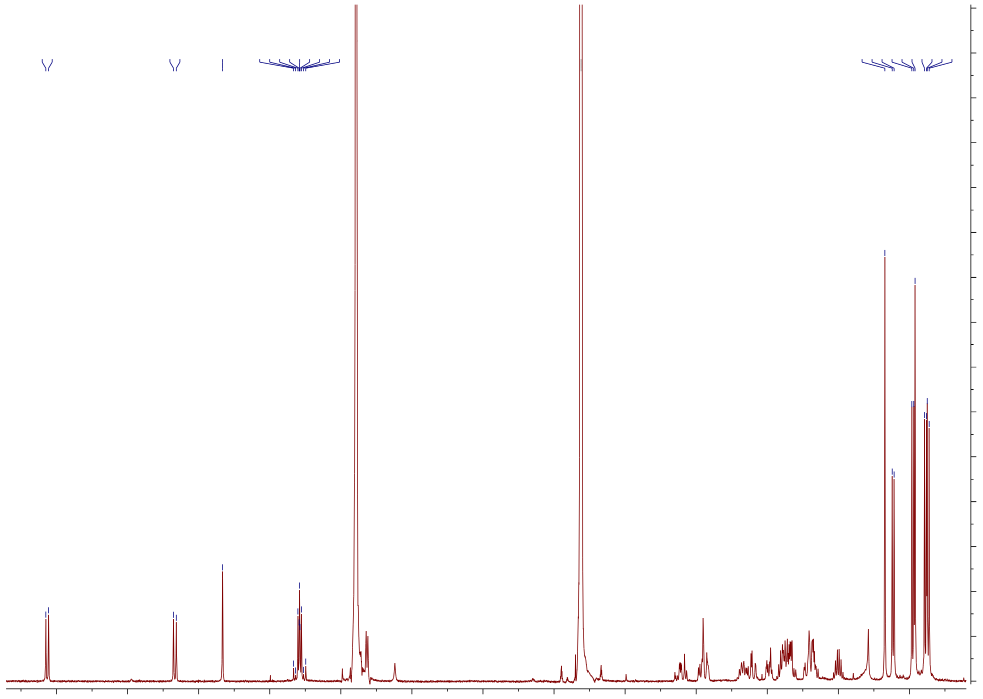

^{1}H-NMR spectrum of compound **2** in CD_3OD

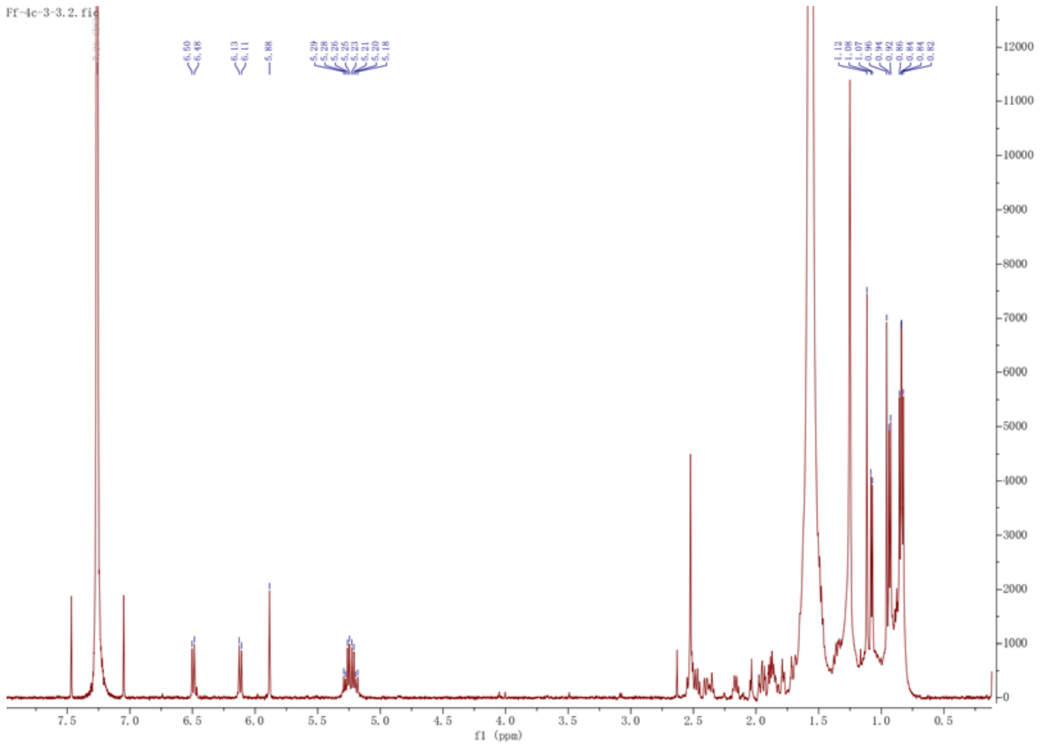

^{1}H-NMR spectrum of compound **3** in $CDCl_3$

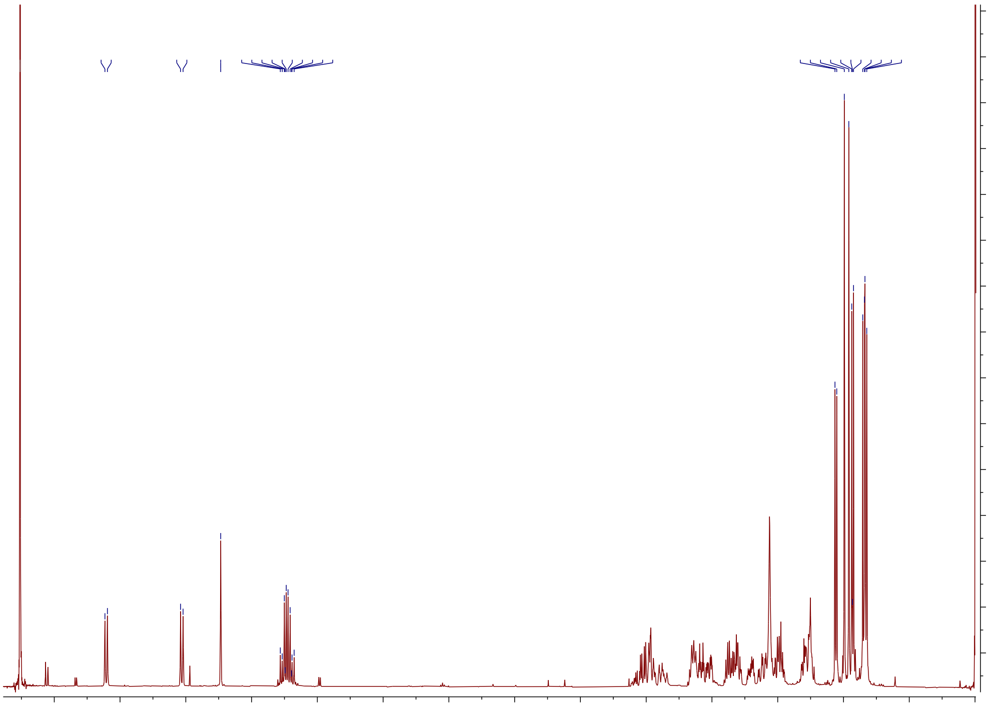

^{1}H-NMR spectrum of compound **4** in $CDCl_3$

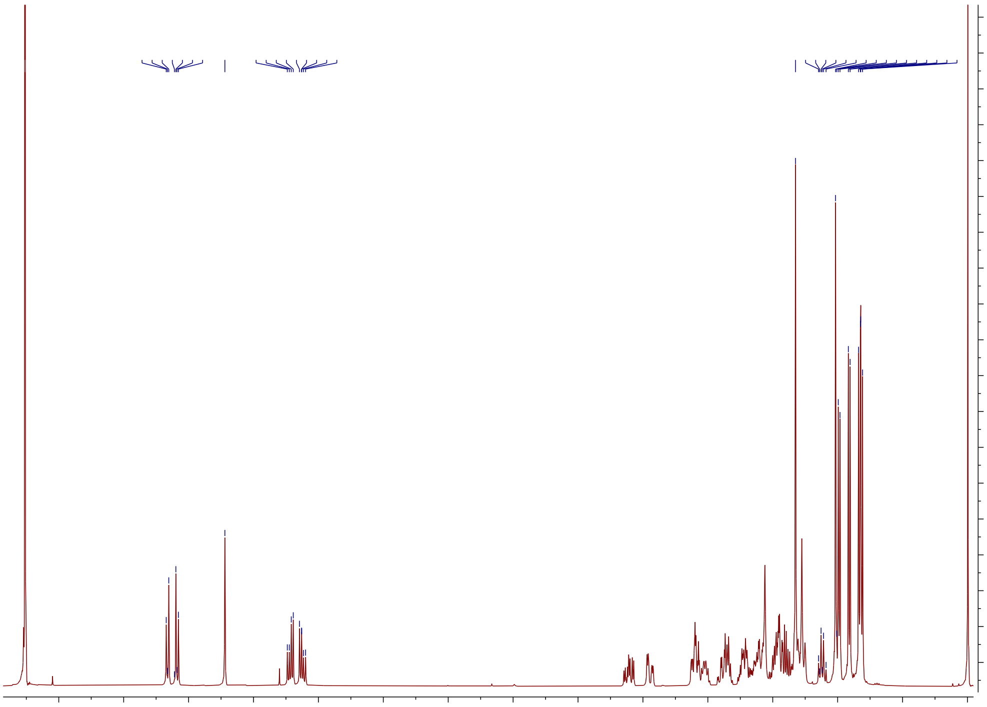

^{1}H-NMR spectrum of compound **5** in $CDCl_3$

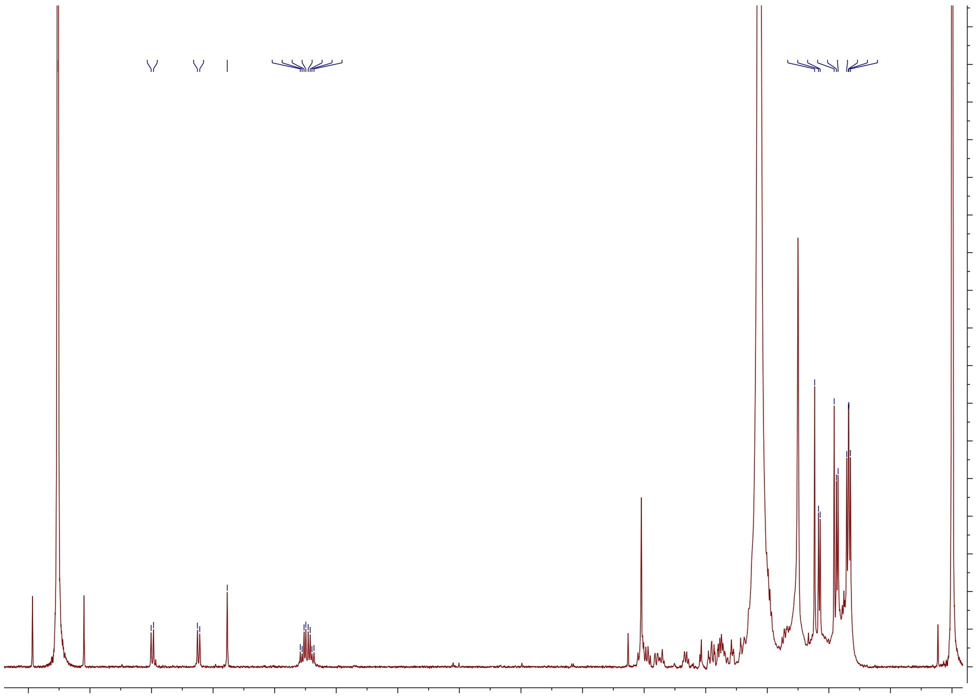

^{1}H-NMR spectrum of compound **6** in $CDCl_3$

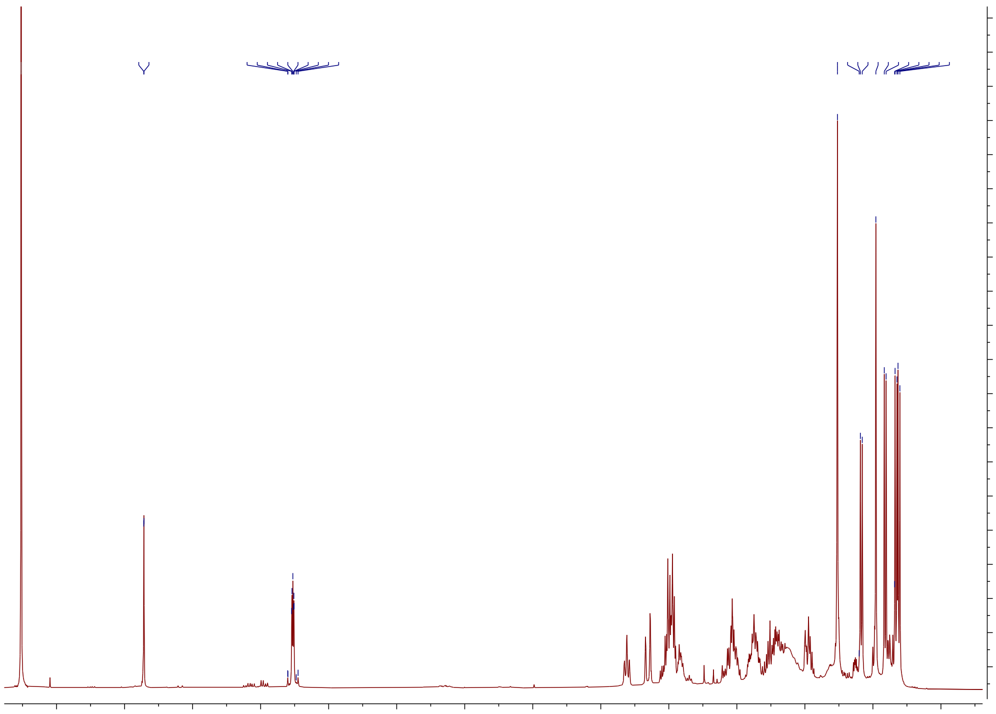

^{1}H-NMR spectrum of compound **7** in $CDCl_3$

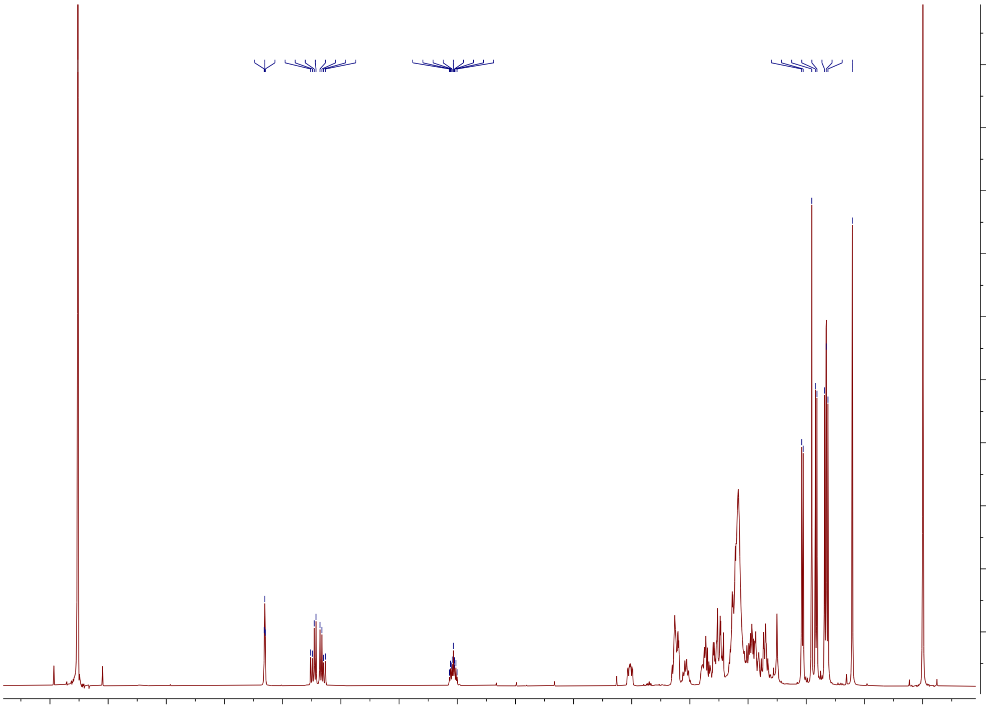

^{1}H-NMR spectrum of compound **8** in $CDCl_3$

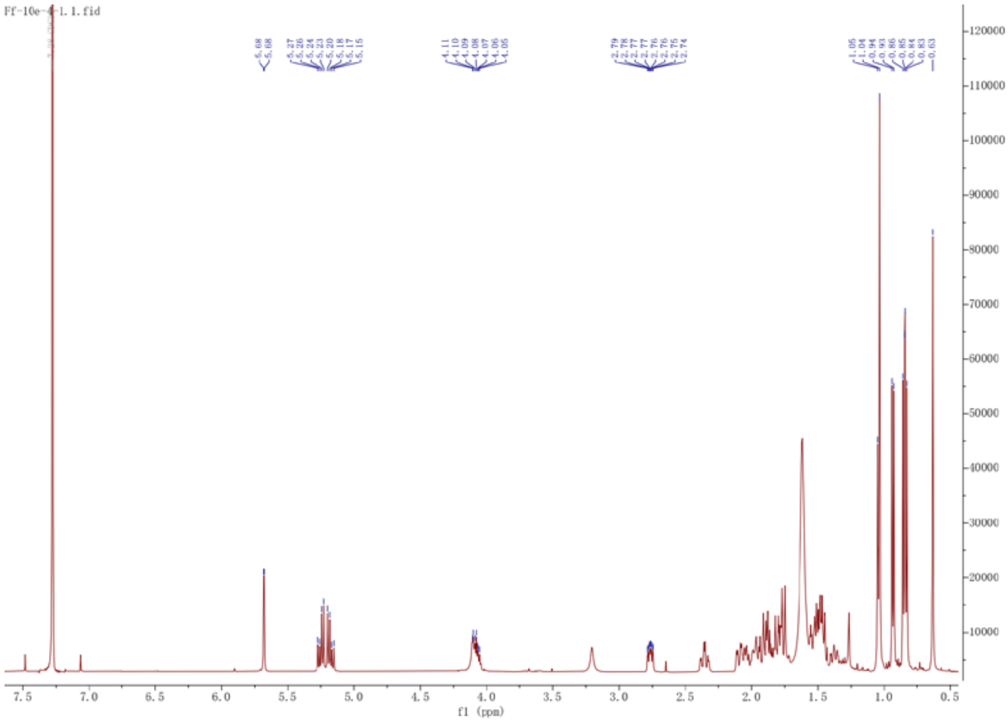

^{1}H-NMR spectrum of compound **9** in $CDCl_3$

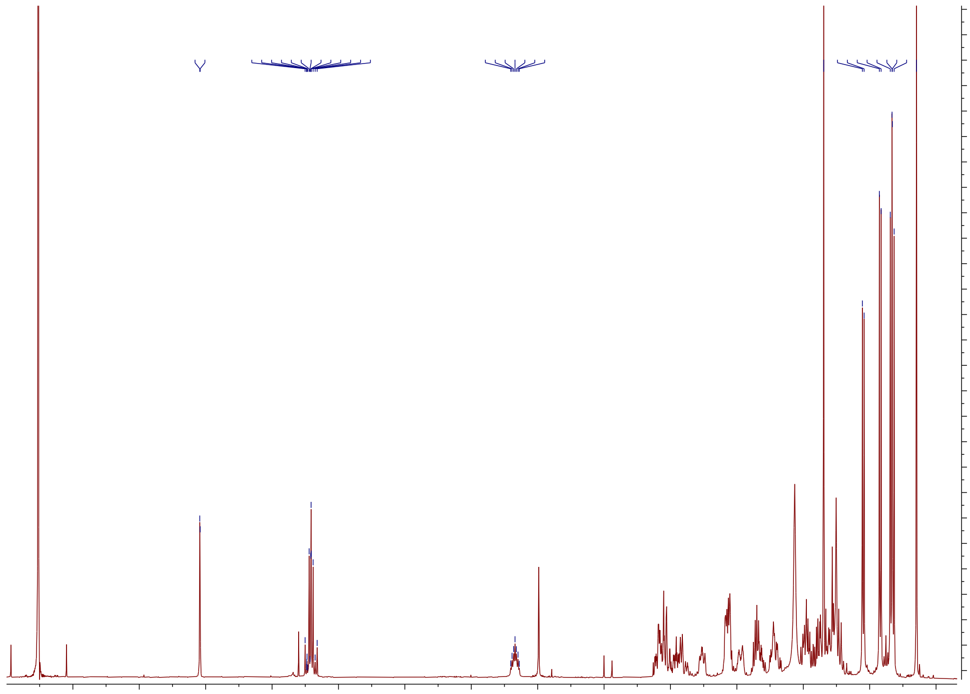

^{1}H-NMR spectrum of compound **10** in $CDCl_3$

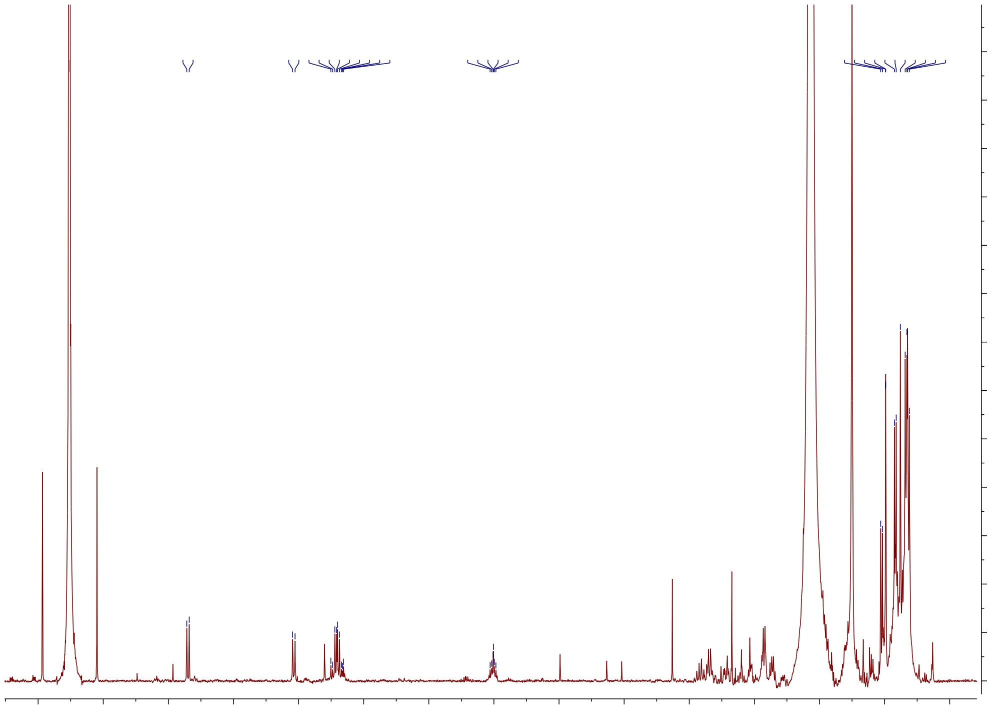

^{1}H-NMR spectrum of compound **11** in $CDCl_3$

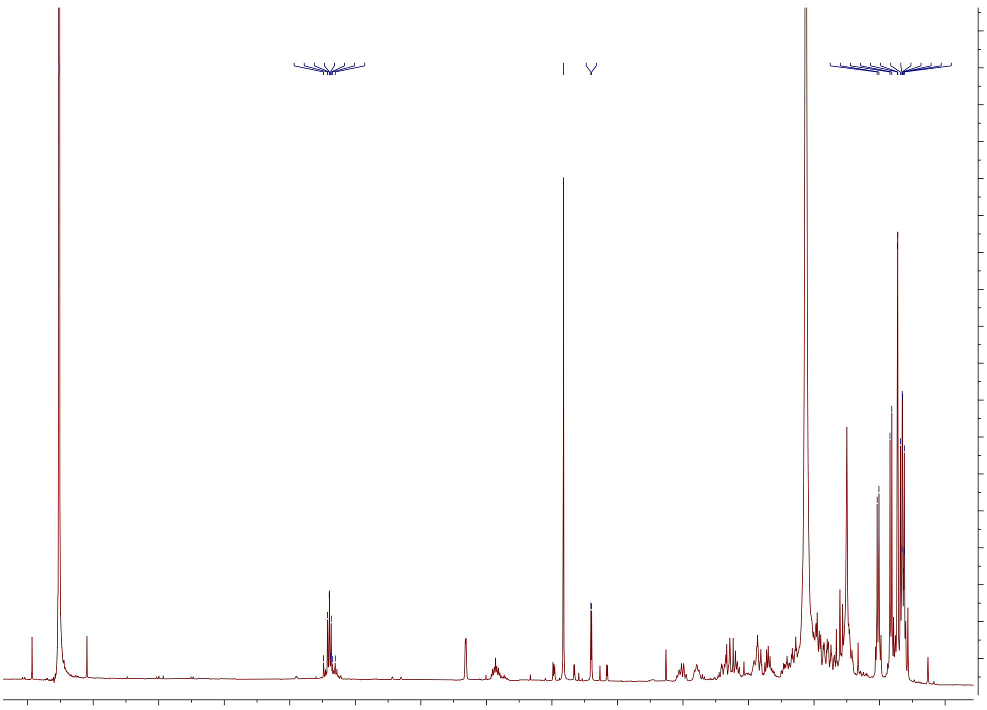

^{1}H-NMR spectrum of compound **12** in $CDCl_3$

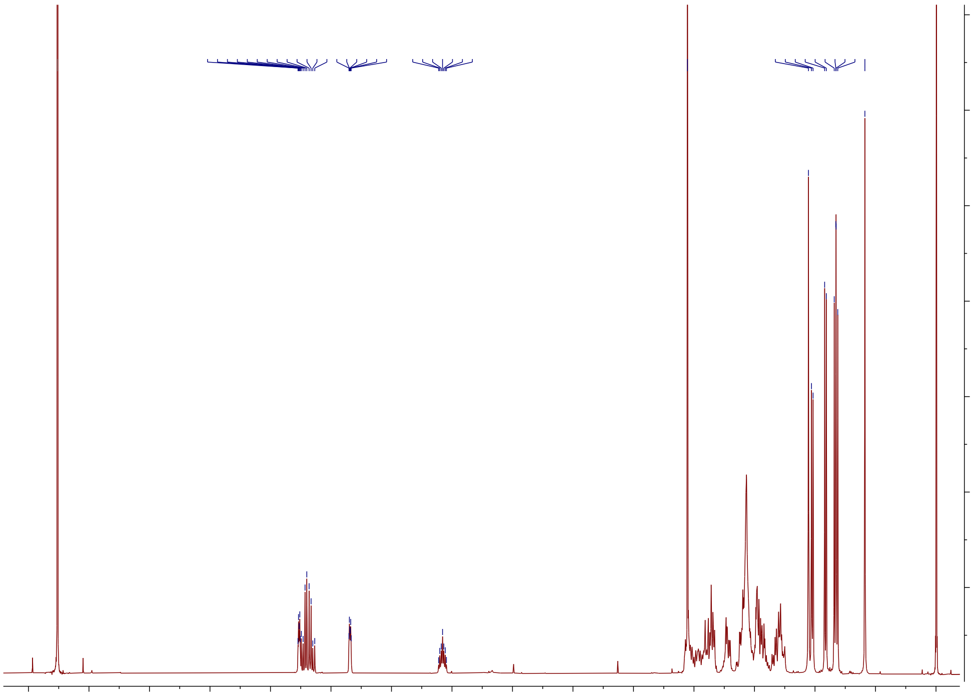

^{1}H-NMR spectrum of compound **13** in $CDCl_3$

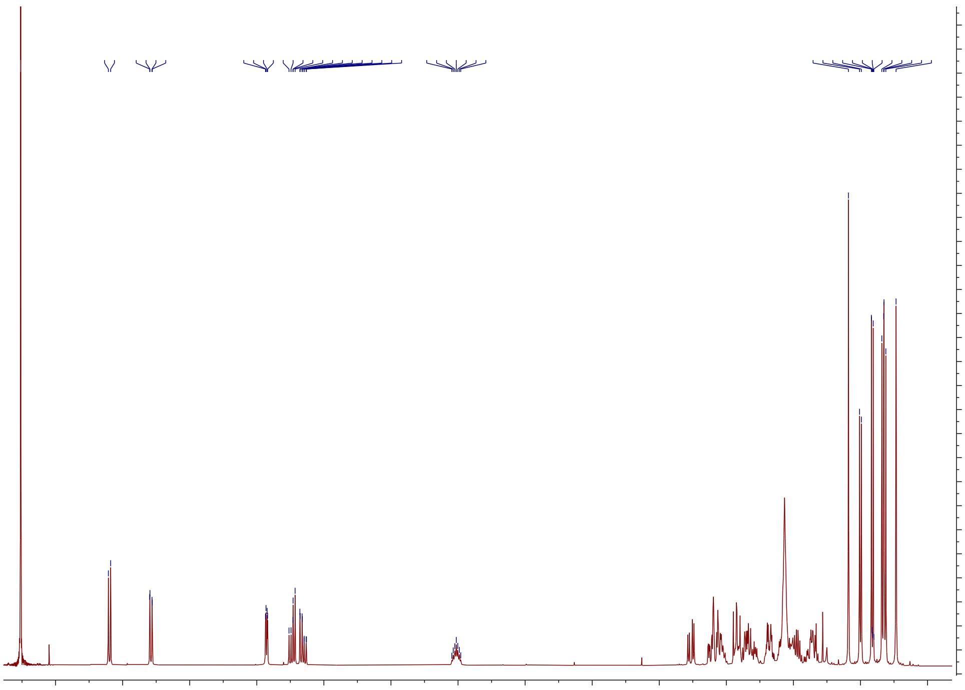

^{1}H-NMR spectrum of compound **14** in $CDCl_3$

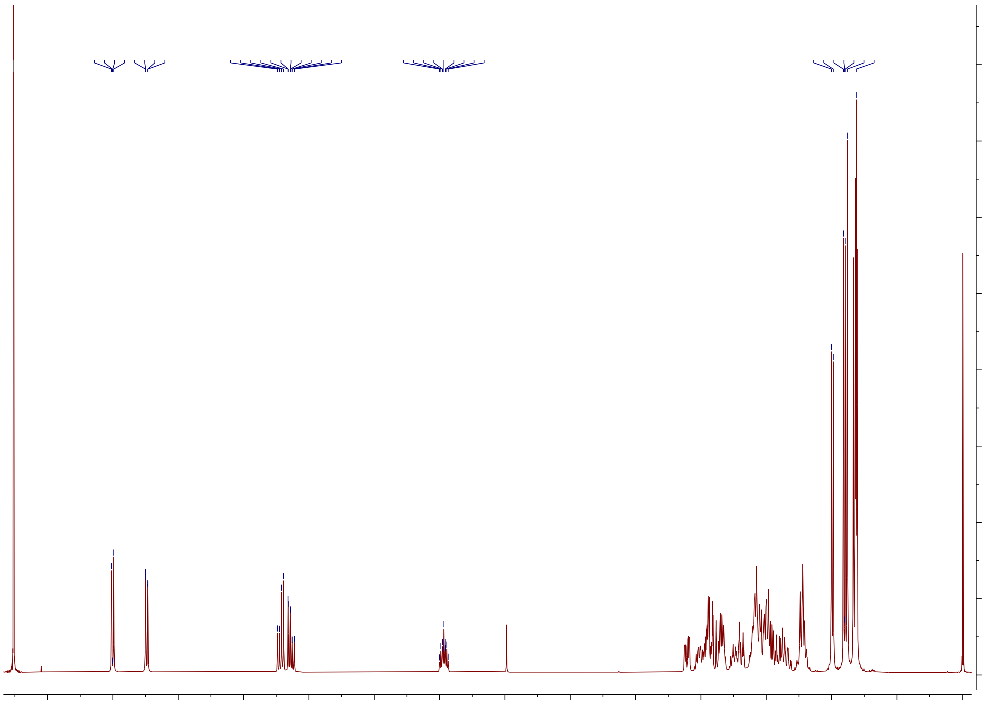

^{1}H-NMR spectrum of compound **15** in $CDCl_3$

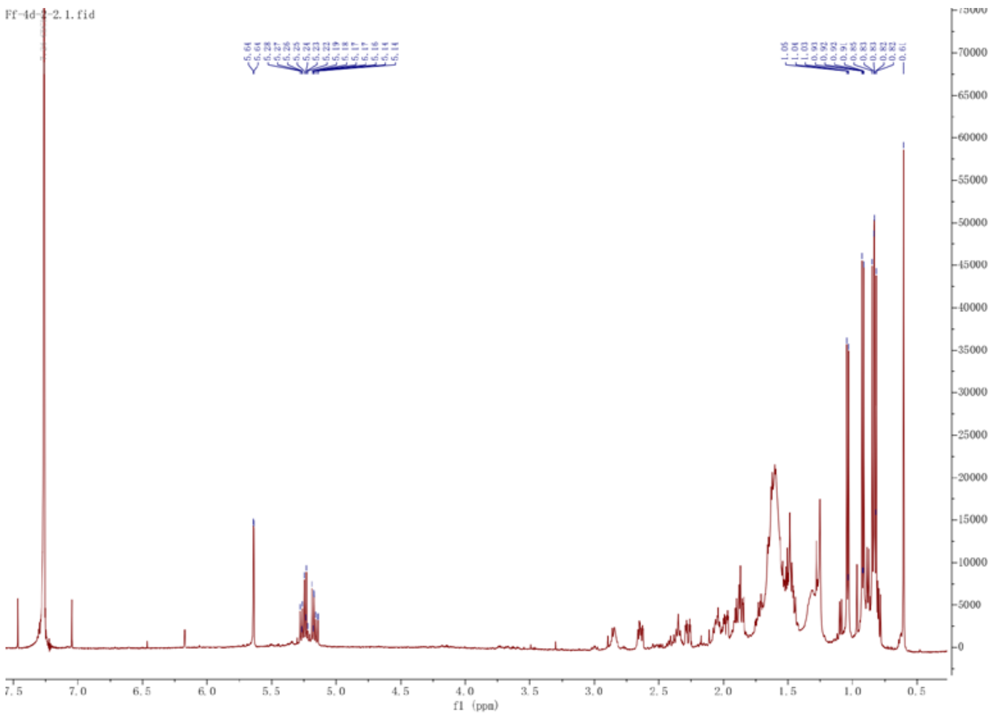

^{1}H-NMR spectrum of compound **16** in $CDCl_3$
